# Avian influenza overview March–May 2026

**DOI:** 10.2903/j.efsa.2026.10196

**Published:** 2026-07-08

**Authors:** European Food Safety Authority (EFSA), European Centre for Disease Prevention and Control (ECDC), European Union Reference Laboratory for Avian Influenza (EURL), Hubert Buczkowski, Mariette Ducatez, Alice Fusaro, Jose L Gonzales, Thijs Kuiken, Gražina Mirinavičiūtė, Karl Ståhl, Christoph Staubach, Olov Svartström, Calogero Terregino, Katriina Willgert, Evelyn Alarcón, Lisa Kohnle

**Keywords:** avian influenza, captive birds, HPAI, humans, monitoring, poultry, wild birds

## Abstract

Between 28 February and 4 June 2026, 949 highly pathogenic avian influenza (HPAI) A(H5) virus detections were reported in domestic (186) and wild (763) birds in 30 countries in Europe. The downward trend in the number of detections observed at the end of the previous reporting period continued and is expected to persist throughout the summer. While the number of HPAI A(H5N1) outbreaks in domestic birds remained at a low level, except in a few countries, A(H9N2) virus of clade G5.5 was detected in poultry in Europe for the first time. Following the intense circulation of HPAI viruses in waterfowl in recent months, sporadic detections were reported in mammals, particularly in wild carnivores, including the detection of A(H5N5) virus in a polar bear and a walrus in Norway. Outside Europe, the focus of HPAI virus detections shifted from North to South America, where a large number of outbreaks and mortality events in swans were reported. Between 28 February and 4 June 2026, 19 cases of avian influenza virus infection were publicly reported in humans (including three fatal cases) in six countries and territories: Bangladesh (two cases with A(H5N1), one fatal), Cambodia (three cases with A(H5N1), one fatal), India (one case with A(H5N1)), Italy (one imported case with A(H9N2)), China (10 A(H9N2) cases and one fatal A(H5N6) case), and Taiwan (one A(H7N7) case). Most human cases reported exposure to poultry or a poultry environment prior to detection or onset of illness. Human infections with avian influenza viruses remain rare and no sustained human‐to‐human transmission has been documented. The risk posed by avian influenza A(H5N1) clade 2.3.4.4b viruses currently circulating in Europe remains low for the general public in the European Union/European Economic Area (EU/EEA) and low‐to‐moderate for those occupationally or otherwise exposed to infected animals or contaminated environments.

**Requestor:** European Commission

**Question number:** EFSA-Q-2026-00136 and Commission request 280 to ECDC (SANTE.B.2/IK/mo (2023)2182203)

**Acknowledgements:** In addition to the listed authors, EFSA, ECDC and the EURL wish to thank the Member State representatives who provided epidemiological data on avian influenza outbreaks or shared sequence data: Lika Aldin and Erga Shkodra (Albania); Sandra Revilla-Fernández and Irene Zimpernik (Austria); Ingeborg Mertens, Mieke Steensels and Steven Van Borm (Belgium); Emiliya Ivanova (Bulgaria); Vladimir Savic (Croatia); Vasiliki Christodoulou (Cyprus); Tomáš Jarosil, Klára Jelínková, Petr Kučínský, Alexander Nagy, Hynek Siksta and Ludek Zavada (Czechia); Charlotte Kristiane Hjulsager, Yuan Liang, Pernille Dahl Nielsen and Heidi Huus Petersen (Denmark); Imbi Nurmoja (Estonia); Tuija Gadd, Ari Kauppinen, Niina Tammiranta and Tiia Tuupanen (Finland); François-Xavier Briand, Thomas Gayen, Béatrice Grasland, Éric Niquex, Audrey Schmitz and Angela Srour (France); Timm Harder, Anne Pohlmann and Carola Sauter-Louis (Germany); Georgina Helyes and Malik Peter (Hungary); Brigitte Brugger and Vilhjálmur Svansson (Iceland); Laura Garza Cuartero (Ireland); Lara Cavicchio, Marta Dianati, Isabella Monne, Angela Salomoni and Bianca Zecchin (Italy); Juris Kibilds, Marta Lizuma and Žanete Šteingolde (Latvia); Simona Pileviciene and Egidijus Pumputis (Lithuania); Vicky Binck, Manon Bourg, Guillaume Fournier and Chantal Snoeck (Luxembourg); Oxana Groza (Moldova); Monika Ballmann, Dennis Bol, Marc Engelsma and Marcel Spierenburg (Netherlands); Aleksandar Dodovski and Igor Dzadzovski (North Macedonia); Anne Døsen, Britt Gjerset and Ingeborg Slettebø Wathne (Norway); Katarzyna Domańska-Blicharz, Anna Pikula, Aleksandra Podrażka, Magdalena Roman and Krzysztof Śmietanka (Poland); Margarida Duarte, Teresa Fagulha and Margarida Henriques (Portugal); Iuliana Onita (Romania); Dejan Vidanovic (Serbia); Zuzana Dirbakova and Martin Tinak (Slovakia); Brigita Slavec (Slovenia); Montserrat Agüero García and María Teresa Barrios Zambrano (Spain); Linnea Lindgren Kero and Siamak Zohari (Sweden); Claudia Bachofen (Switzerland); Michael McMenemy and colleagues from the Department of Agriculture, Environment and Rural Affairs (DAERA) (Northern Ireland)[1]). The authors also wish to thank Ron Fouchier from Erasmus Medical Center (Netherlands); Ashley Banyard and Holly Coombes from the Animal and Plant Health Agency (United Kingdom); Denys Muzyka from the National Scientific Center Institute of Experimental and Clinical Veterinary Medicine (Ukraine); Camille Delavenne from EpiMundi for conducting the data analysis under the contract EFSA/2025/OP/0014; the authors, originating and submitting laboratories of the sequences from GISAID's EpiFlu™ Database, which is used for this assessment (Annex E); Julio Álvarez from EFSA's Panel on Animal Health and Welfare (AHAW); Edoardo Colzani from ECDC, as well as Miguel Melo and Stefania Tampach from EFSA for the support provided to this scientific output.

**Suggested citation:** EFSA (European Food Safety Authority), ECDC (European Centre for Disease Prevention and Control), EURL (European Union Reference Laboratory for Avian Influenza), Buczkowski, H., Ducatez, M., Fusaro, A., Gonzalez, J. L., Kuiken, T., Mirinavičiūtė, G., Ståhl, K., Staubach, S., Svartström, O., Terregino, C., Willgert, K., Alarcón, A., & Kohnle, L.

[1] In accordance with the Agreement on the Withdrawal of the United Kingdom from the EU, and in particular with the Windsor Framework, the EU requirements on data sampling are also applicable to the United Kingdom (Northern Ireland).

(2026). Avian influenza overview March–May 2026. *EFSA Journal*, *24*(7), e10196, https://doi.org/10.2903/j.efsa.2026.10196

**ISSN:** 1831-4732

Reproduction is authorised provided the source is acknowledged.

EFSA may include images or other content for which it does not hold copyright. In such cases, EFSA indicates the copyright holder and users should seek permission to reproduce the content from the original source.

**Map disclaimer:** The designations employed and the presentation of material on any maps included in this scientific output do not imply the expression of any opinion whatsoever on the part of the European Food Safety Authority concerning the legal status of any country, territory, city or area or of its authorities, or concerning the delimitation of its frontiers or boundaries.

## Table of contents

Abstract ........ 1
Table of contents ........ 4
1 Introduction ........ 5
2 Assessment ........ 5
2.1 HPAI virus detections in birds ........ 5
2.1.1 HPAI virus detections in birds in Europe ........ 5
2.1.2 HPAI virus detections in birds outside Europe ........ 19
2.1.3 Genetic characteristics of HPAI viruses of the A(H5Nx) subtype in avian species ........ 24
2.1.4 Genetic characteristics of avian influenza viruses of other subtypes in avian species ........ 27
2.2 HPAI virus detections in non-human mammals ........ 27
2.2.1 Genetic characteristics of HPAI viruses of the A(H5Nx) subtype circulating in Europe in non-human mammals ........ 32
2.3 Avian influenza virus infections in humans ........ 33
2.3.1 Overview of the most recent human infections with avian influenza viruses ........ 33
2.3.2 Human A(H5N1) cases ........ 35
2.3.3 Human A(H5N6) cases ........ 37
2.3.4 Human A(H7N7) cases ........ 38
2.3.5 Human A(H9N2) cases ........ 39
2.3.6 Additional information relevant for public health and international risk assessments ........ 40
2.3.7 ECDC risk assessment ........ 41
3 Summary and conclusions ........ 43
3.1 Birds ........ 43
3.2 Mammals ........ 44
3.3 Humans ........ 44
4 Options for response ........ 45
4.1 Birds ........ 45
4.2 Mammals ........ 46
4.3 Humans ........ 46
References ........ 49
Appendix A – Terms of Reference of the joint EFSA-ECDC mandate (M-2024-00009) accepted in March 2024 ........ 63
Appendix B – Data and Methodologies ........ 65
Annex A – Data on HPAI virus detections in birds ........ 67
Annex B – Characteristics of the HPAI-affected poultry establishments ........ 67
Annex C – Data on HPAI virus detections in mammals ........ 67
Annex D – General options for response in animals ........ 67
Annex E – Acknowledgements ........ 67

## 1 Introduction

This Scientific Report provides an overview of highly pathogenic avian influenza (HPAI) virus detections in poultry[2] and captive birds[3] (domestic birds), as well as in wild birds, that occurred in and outside Europe between 28 February and 4 June 2026. In addition, the report includes HPAI virus detections in mammals up until 22 May 2026 and cases of avian influenza infection in humans between 28 February and 4 June 2026. Detections of low pathogenic avian influenza (LPAI) virus in birds are discussed whenever they are of zoonotic concern or otherwise relevant.

The background, Terms of Reference (TOR), and interpretation thereof are described in Appendix A, whereas the data and methodologies used are reported in Appendix B.

## 2 Assessment

### 2.1 HPAI virus detections in birds

#### 2.1.1 HPAI virus detections in birds in Europe

Figure 1 shows all HPAI virus detections in birds in Europe that were reported via the European Union (EU) Animal Disease Information System (ADIS) or the World Animal Health Information System (WOAH-WAHIS) of the World Organisation for Animal Health (WOAH) for the last six and the current epidemiological year[4] by month of suspicion. For the current epidemiological year 2025–2026, starting on 1 October 2025, data reported are truncated on 4 June 2026.

[2] According to Regulation (EU) 2016/429 of the European Parliament and of the Council of 9 March 2016, point (9), 'poultry' means birds that are reared or kept in captivity for: (a) the production of: (i) meat; (ii) eggs for consumption; (iii) other products; (b) restocking supplies of game birds; (c) the purpose of breeding of birds used for the types of production referred to in points (a) and (b).
[3] According to Regulation (EU) 2016/429 of the European Parliament and of the Council of 9 March 2016, point (10), 'captive birds' means any birds other than poultry that are kept in captivity for any reason other than those referred to in point (9), including those that are kept for shows, races, exhibitions, competitions, breeding or selling.
[4] In this document an 'epidemiological year' refers to the period starting on 1 October and ending on 30 September of the following year, based on the dates on which the first HPAI virus detections were observed in wild birds in Europe in 2016–2017, 2020–2021 and 2021–2022.

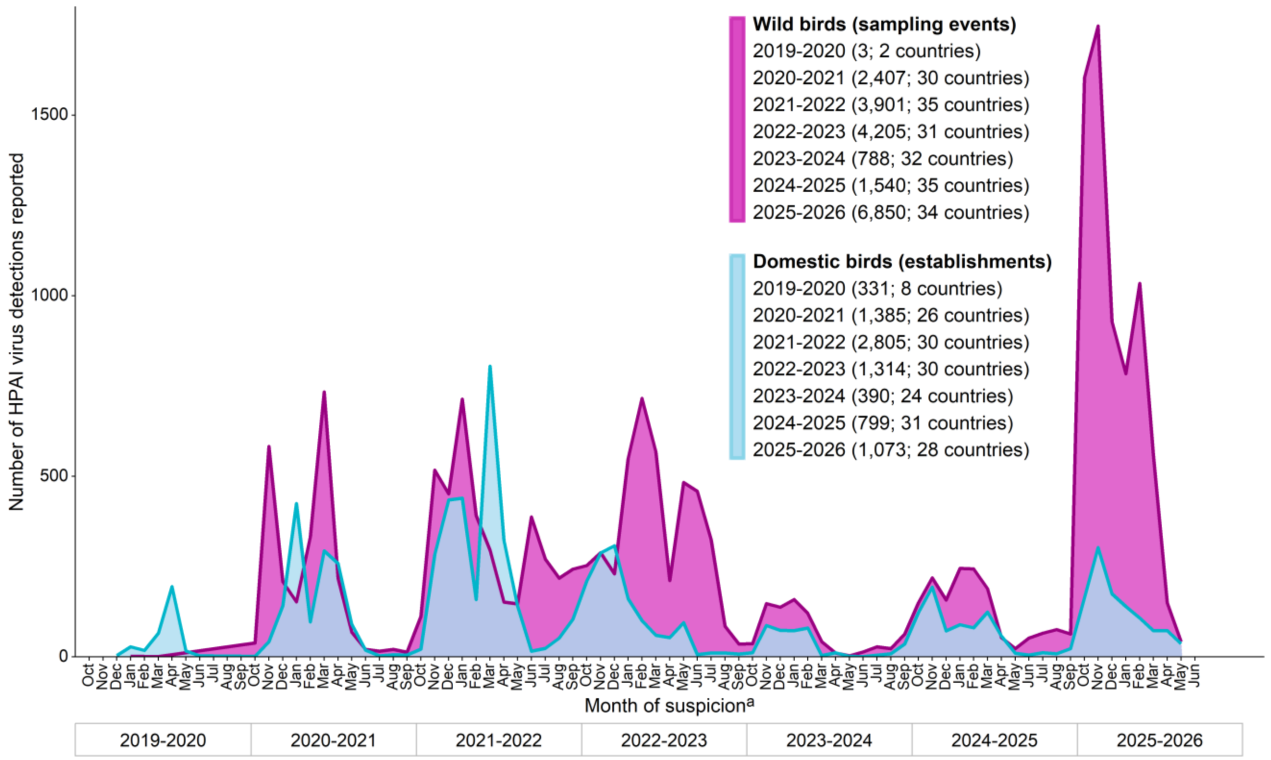

Figure 1: Distribution of the number of HPAI virus detections in wild birds (cumulative number n = 19,694) (pink) and establishments keeping domestic birds (cumulative n = 8097) (blue) reported in Europe during seven epidemiological years by month of suspicion, from 1 October 2019 to 4 June 2026 (total n = 27,791).

*Note*: United Kingdom data are from the Animal Disease Notification System (ADNS, former ADIS) up until 31 December 2020. From 1 January 2021 onwards, the data source was WOAH-WAHIS for the United Kingdom (excluding Northern Ireland) and ADNS/ADIS for the United Kingdom (Northern Ireland)[5].

[a] If the date of suspicion was not available, the date of confirmation was used to assign the week of suspicion.

*Source*: ADNS/ADIS and WOAH-WAHIS (data extraction carried out on 4 June 2026).

Considering the current reporting period from 28 February to 4 June 2026, 949 HPAI virus detections were reported in poultry (154), captive (32) and wild (763) birds in 30 countries in Europe (Table 1, Figure 2).

[5] In accordance with the Agreement on the Withdrawal of the United Kingdom from the EU, and in particular with the Windsor Framework, the EU requirements on data sampling are also applicable to the United Kingdom (Northern Ireland).

Table 1: Number of HPAI outbreaks reported in Europe by country, virus subtype and affected sub-population, from 28 February to 4 June 2026. Cumulative numbers since the start of the 2025-2026 epidemiological year are reported in parentheses (1 October 2025 to 4 June 2026).

| Country | Captive birds | Poultry | | | Wild birds | | | | Total |
|---|---|---|---|---|---|---|---|---|---|
| | A(H5N1) | A(H5Nx) | A(H5N1) | A(Not typed) | A(H5Nx) | A(H5N1) | A(H5N2) | A(H5N5) | |
| Austria | 0 (4) | - | 0 (1) | - | - | 30 (91) | - | - | 30 (96) |
| Belgium | 0 (3) | - | 0 (20) | - | 5 (47) | 16 (333) | - | - | 21 (403) |
| Bosnia and Herzegovina | - | - | 0 (1) | - | - | 0 (1) | - | - | 0 (2) |
| Bulgaria | 0 (1) | - | 2 (15) | - | - | 1 (1) | - | - | 3 (17) |
| Croatia | - | - | - | - | - | 1 (7) | - | - | 1 (7) |
| Czechia | 7 (35) | - | 6 (20) | - | - | 3 (44) | - | - | 16 (99) |
| Denmark | 2 (6) | - | 9 (25) | - | - | 66 (225) | - | 0 (1) | 77 (257) |
| Estonia | 1 (4) | - | - | - | - | 15 (29) | - | - | 16 (33) |
| Finland | - | - | - | 1 (2) | 0 (1) | 11 (37) | - | - | 12 (40) |
| France | 2 (31) | - | 6 (125) | - | - | 5 (310) | - | - | 13 (466) |
| Germany | 13 (71) | - | 7 (199) | - | - | 403 (3763) | - | - | 423 (4033) |
| Greece | - | - | - | - | - | 1 (1) | - | - | 1 (1) |
| Hungary | - | - | 3 (16) | - | - | 3 (55) | - | - | 6 (71) |
| Iceland | - | - | - | - | 0 (1) | 0 (4) | - | 2 (3) | 2 (8) |
| Ireland | 0 (1) | - | 0 (5) | - | - | 1 (12) | - | - | 1 (18) |
| Italy | 0 (2) | - | 3 (65) | - | 0 (13) | 1 (97) | - | - | 4 (177) |
| Latvia | 0 (2) | - | - | - | - | 1 (27) | 0 (1) | - | 1 (30) |
| Lithuania | - | - | 0 (3) | - | - | 2 (30) | - | - | 2 (33) |
| Luxembourg | 0 (1) | - | - | - | - | 0 (23) | - | - | 0 (24) |
| Moldova | - | - | - | - | - | 2 (7) | - | - | 2 (7) |
| Montenegro | - | - | 4 (5) | - | - | - | - | - | 4 (5) |
| Netherlands | 0 (14) | - | 7 (46) | - | - | 35 (372) | - | - | 42 (432) |
| North Macedonia | 0 (2) | - | - | - | - | - | - | - | 0 (2) |
| Norway | - | - | - | - | 0 (9) | 17 (65) | - | - | 17 (74) |
| Poland | 6 (20) | - | 102 (182) | - | - | 51 (256) | - | - | 159 (458) |
| Portugal | 0 (4) | 0 (1) | 0 (11) | - | - | 1 (11) | - | - | 1 (27) |
| Romania | 0 (1) | - | 0 (1) | - | - | 3 (12) | - | - | 3 (14) |
| Serbia | - | - | - | - | - | 0 (1) | - | - | 0 (1) |
| Slovakia | 0 (4) | - | 0 (4) | - | - | 0 (11) | - | - | 0 (19) |
| Slovenia | - | - | - | - | - | 1 (18) | - | - | 1 (18) |
| Spain | 0 (4) | - | 0 (7) | - | 0 (1) | 2 (115) | - | - | 2 (127) |
| Sweden | 0 (2) | - | 0 (7) | - | - | 21 (112) | 0 (1) | - | 21 (122) |
| Switzerland | 0 (1) | - | - | - | - | 0 (24) | - | - | 0 (25) |
| Ukraine | 1 (2) | - | - | - | - | 0 (2) | - | - | 1 (4) |
| United Kingdom (excluding Northern Ireland) | - | - | 1 (4) | - | - | 0 (6) | - | - | 1 (10) |

| | | | | | | | | | |
|---|---|---|---|---|---|---|---|---|---|
| United Kingdom (Northern Ireland)[a] | 0 (18) | - | 4 (77) | - | - | 62 (665) | - | 0 (3) | 66 (763) |
| **Total** | **32 (233)** | **0 (1)** | **154 (839)** | **1 (2)** | **5 (72)** | **755 (6767)** | **0 (2)** | **2 (7)** | **949 (7923)** |

–, no HPAI outbreaks were notified via ADIS or WOAH-WAHIS.

[a] In accordance with the Agreement on the Withdrawal of the United Kingdom from the EU, and in particular with the Windsor Framework, the EU requirements on data sampling are also applicable to the United Kingdom (Northern Ireland).

*Source*: ADIS and WOAH-WAHIS (data extraction carried out on 4 June 2026).

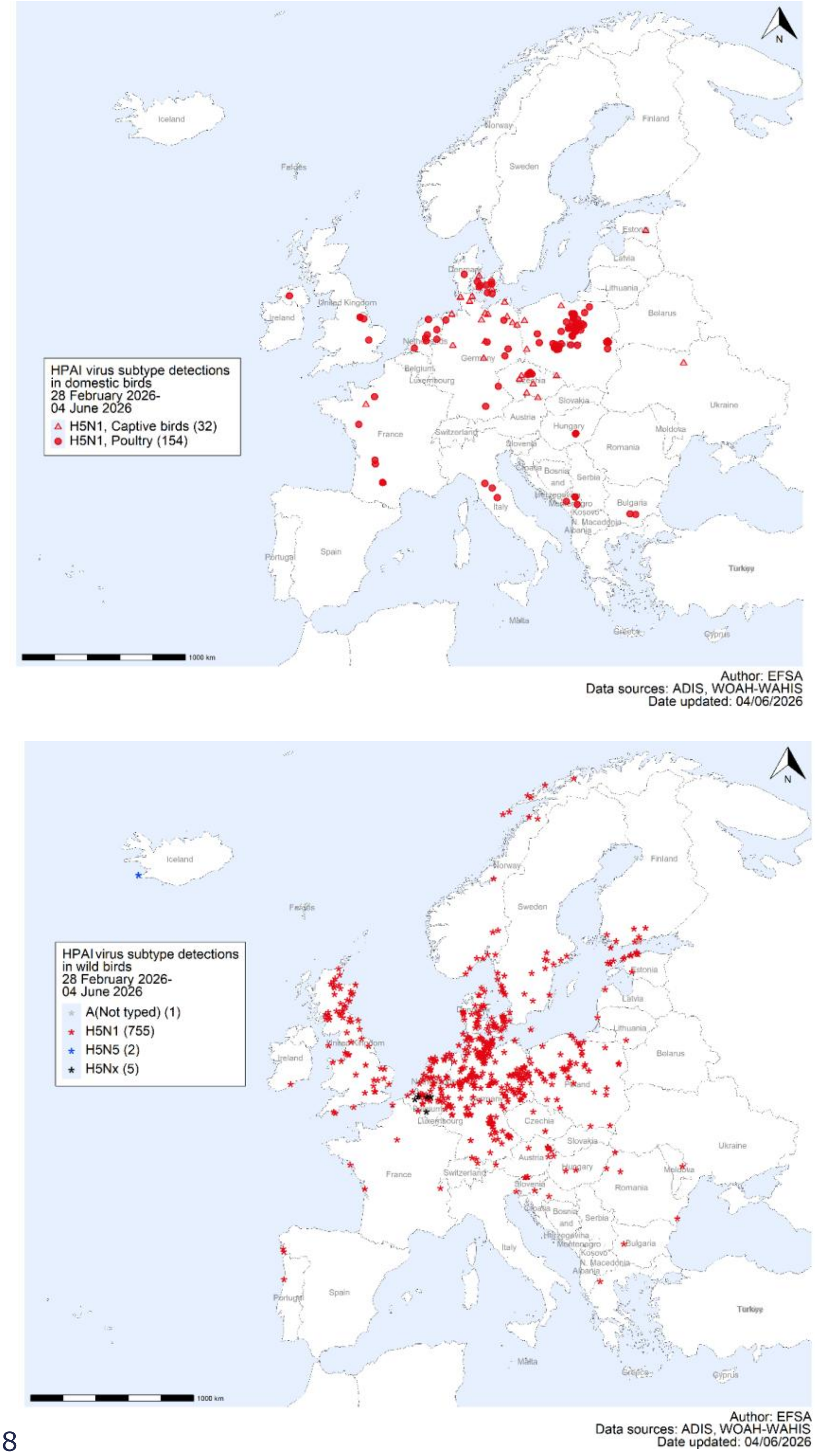

8

Figure 2: Geographic distribution, based on available geocoordinates, of HPAI virus detections in poultry and captive birds (186) (upper panel), and in wild birds (763) (lower panel), reported by virus subtype in Europe from 28 February to 4 June 2026.

* This designation is without prejudice to positions on status and is in line with United Nations Security Council Resolution 1244 and the International Court of Justice Opinion on the Kosovo Declaration of Independence.

*Source*: ADIS and WOAH-WAHIS (data extraction carried out on 4 June 2026).

The observed decline in the number of HPAI virus detections in Europe, which had started in the previous reporting period, continued throughout spring, with a sharper decline in wild birds than in domestic birds (Figure 1, Table 1). However, a second peak in detections is clearly marked at the beginning of the current reporting period, thereby continuing the two-peak pattern observed in wild birds in previous epidemiological years. Compared to the same period last year, the number of detections in domestic birds was similar, while the number of detections in wild birds in the 2025–2026 epidemiological year was at least three times higher, due to the very high circulation of HPAI virus in waterfowl.

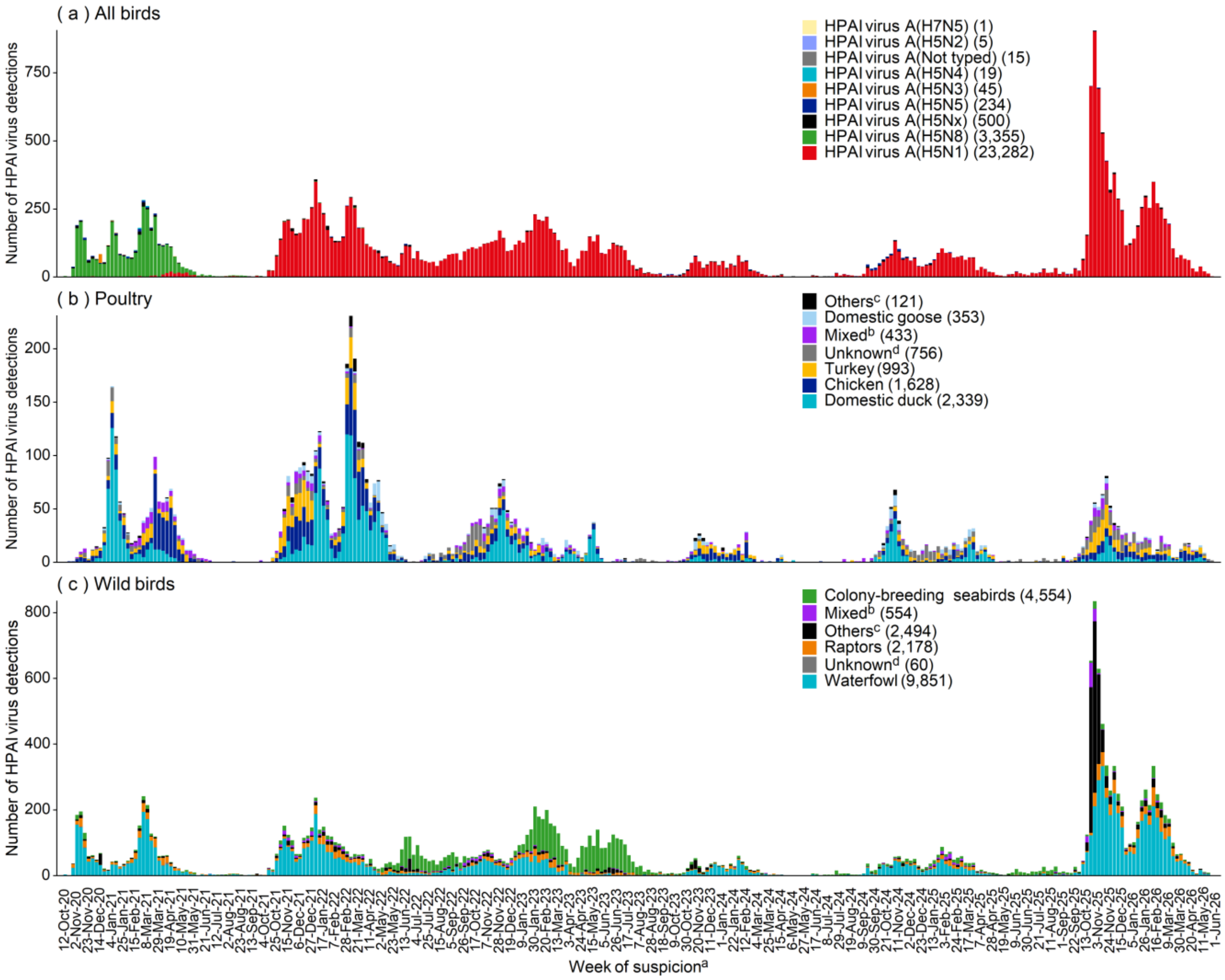

Figure 3: Distribution of the total number of HPAI virus detections reported in Europe by week of suspicion (dates indicate the first day of the week) and virus subtype (a), affected poultry categories (b), and affected wild bird categories (c), from 1 October 2020 to 4 June 2026.

[a] If the date of suspicion was not available, the date of confirmation was used to assign the week of suspicion.
[b] Mixed, outbreaks in which multiple species or categories were involved.
[c] Others, all other affected categories which are not indicated in the legend.
[d] Unknown, affected categories which were not further specified during reporting.

*Source*: ADNS/ADIS, EFSA and WOAH-WAHIS (data extraction carried out on 4 June 2026).

The temporal and geographical distributions of HPAI outbreaks in domestic birds (Figure 2, upper panel; Figure 3b) were only partly correlated with those of HPAI virus detections in wild birds, which were far more widespread (Figure 2, lower panel; Figure 3c). In the current reporting period, HPAI in poultry took the form of a smouldering epidemic in the vast majority of European countries reporting outbreaks. This was followed by a marked decline in the total number of outbreaks compared to the winter months. Transmission (either from wild birds to poultry or between poultry flocks) persisted and was sustained locally, particularly in Poland (Annex B), where the highest number of outbreaks in poultry was reported (Table 1). HPAI virus detections in wild birds showed a continued sharp decline in numbers throughout the current reporting period and were concentrated in northern and central-western parts of Europe, from the United Kingdom (excluding Northern Ireland) in the west to Estonia in the east, and from northern Norway to Austria in the south. In addition, there were sporadic detections on the Iberian Peninsula in the southwest and in southeastern Europe, such as in Greece. The vast majority of HPAI viruses detected in domestic and wild birds belonged to subtype A(H5N1) (Table 1; Figure 3c), although two HPAI A(H5N5) virus detections were also reported in Iceland (Figure 2, lower panel).

Spatio-temporal information on all HPAI virus detections reported in Europe since October 2016 is available via EFSA's interactive dashboard[6].

### Poultry

Between 28 February and 4 June 2026, 154 HPAI A(H5N1) outbreaks in poultry were reported in 12 countries in Europe: Poland (102), Denmark (9), Germany (7), Netherlands (7), Czechia (6), France (6), Montenegro (4), United Kingdom (excluding Northern Ireland) (4), Hungary (3), Italy (3), Bulgaria (2), and United Kingdom (Northern Ireland)[7] (1) (Table 1, Figure 2).

Poland accounted for 66% (102/154) of these outbreaks (Table 1) and 88% (5,716,654/6,534,773) of the poultry heads culled. In total, more than 6 million birds died or were culled in the HPAI-affected establishments, about 149% less than in the previous reporting period. Between 28 February and 4 June 2026, the most affected poultry category in Europe was the 'chicken' category (31.8%, 49/154 outbreaks), followed by the 'turkey' (23%, 36/154), 'domestic duck' (21%, 32/154), 'domestic goose' (5%, 8/154), and 'other' (1%, 1/154) categories. Multiple species were kept in 9 (6%) establishments, while no information on the species kept was available for another 19 (12%) establishments (Figure 3, Figure 4).

[6] http://hpai.efsa.aus.vet/
[7] In accordance with the Agreement on the Withdrawal of the United Kingdom from the EU, and in particular with the Windsor Framework, the EU requirements on data sampling are also applicable to the United Kingdom (Northern Ireland).

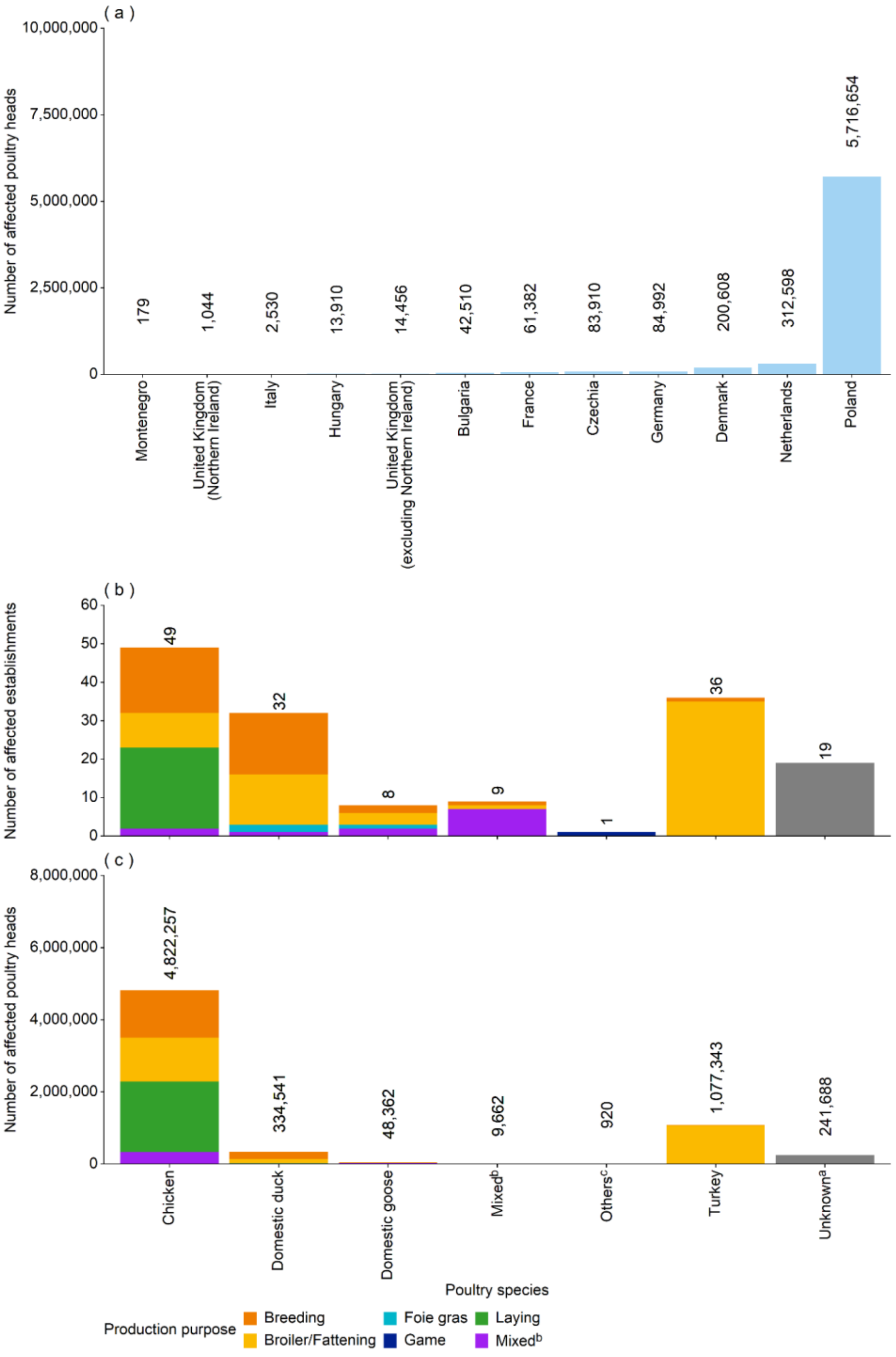

Figure 4: Number of poultry heads in the HPAI-affected establishments per country (total n = 6,534,773; countries are ranked according to the number of poultry heads affected) (a), as well as number of HPAI-affected establishments (total n = 154) (b) and poultry heads (total n = 6,534,773) (c) per poultry species and production purpose in Europe between 28 February and 4 June 2026.

[a] Unknown, affected categories which were not further specified during reporting.
[b] Mixed, outbreaks in which multiple species or categories were involved.
[c] Others, all other affected categories which are not indicated in the legend.

*Source*: ADIS, EFSA and WOAH-WAHIS (data extraction carried out on 4 June 2026).

Within the ‘chicken’ category, 35% (17/49) were chickens for breeding, 43% (21/49) laying hens, 18% (9/49) broilers, and 4% (2/49) chickens for mixed purposes. Within the ‘domestic duck’ category, 50% (16/32) were ducks for breeding, 41% (13/32) ducks for fattening, 6% (2/32) ducks for foie gras production, and 3% (1/32) ducks for mixed purposes (Figure 4).

In the following paragraphs, a brief description of the HPAI outbreaks in poultry is given by country. This description is based on information collected by EFSA from ADIS and WOAH-WAHIS, reporting countries (in form of additional data requested and personal communications), and media reports. Additional data on the characteristics of the affected poultry establishments (e.g. poultry species, production type, source of introduction, number of exposed people, clinical signs and mortality) were collected for 135 of the 154 (88%) outbreaks reported from Czechia, Denmark, France, Germany, Hungary, Netherlands, Poland, and United Kingdom (Northern Ireland)[8] (Annex B). Of these, 73 (47%) indicated indirect contact with wild birds as the most likely source of introduction, while in another 26 (17%) outbreaks, indirect contact with poultry was reported as the likely source, and in one case (1%), direct contact with wild birds was suspected. No information on the source of introduction was available for the remaining 54 (35%) outbreaks. For 19 (12%) outbreaks in poultry, no additional data were collected, either because reporting countries did not submit those data to EFSA or are not reporting via ADIS, or because they occurred too close to the publication of this report. Therefore, only a short summary of those outbreaks will be provided here, while additional data will only be collected in the following round and included in Annex B of the following report.

*Bulgaria*

During the current reporting period from 28 February to 4 June 2026, 2 HPAI A(H5N1) outbreaks in poultry were reported in Bulgaria via ADIS. No additional data to complement the information in ADIS were provided by the reporting country. The first outbreak was reported on 9 March 2026 in an establishment keeping ducks (n = 28,846), where clinical signs were observed and 1482 (5%) dead birds were reported. The second outbreak was reported on 31 March 2026 in another establishment keeping ducks (n = 13,664), where clinical signs were observed and 350 (3%) dead birds were reported. According to the media, both outbreaks occurred in commercial farms in the Haskovo Province (BTA, online).

*Czechia*

During the current reporting period from 28 February to 4 June 2026, 6 HPAI A(H5N1) outbreaks in poultry were reported in Czechia via ADIS. Additional data were collected and provided by the reporting country for all these outbreaks (Annex B). All 6 outbreaks occurred in March in commercial establishments keeping ducks for breeding (total n = 83,910), belonging to the same owner (European Commission, online). Mortality was recorded in all these establishments, with a total of 1369 dead birds involved. Clinical signs were reported in 4 of the 6 outbreaks (67%), whereas no clinical signs were observed in the remaining 2 (33%). A drop in egg production was reported in 5 of the 6 outbreaks (83%), however, this information was not available for the last outbreak. Indirect contact with poultry was indicated as the most likely source of introduction in 4 of the 6 (67%) establishment, while it was indirect contact with wild birds in the other 2 (33%). A total of 135 persons were reported as exposed.

[8] In accordance with the Agreement on the Withdrawal of the United Kingdom from the EU, and in particular with the Windsor Framework, the EU requirements on data sampling are also applicable to the United Kingdom (Northern Ireland).

*Denmark*

During the current reporting period from 28 February to 4 June 2026, 9 HPAI A(H5N1) outbreaks in poultry were reported in Denmark via ADIS. Additional data were collected and provided by the reporting country for all these outbreaks (Annex B). The 9 outbreaks occurred between March and April in both non-commercial (5/9, 56%) and commercial (4/9, 44%) establishments, with a total of 200,608 birds affected. These establishments kept mixed species for mixed purposes (3/9, 33.3%), chickens for breeding (2/9, 22.2%), turkeys for fattening (2/9, 22.2%), pheasants for game purposes (1/9, 11.1%), and mixed species for breeding (1/9, 11.1%). Outdoor access was available in the 5 (56%) non-commercial establishments, keeping mixed species and pheasants, where indirect contact with wild birds was indicated as the most likely source of introduction. Outdoor access was not available in the 4 (44%) commercial establishments, where the source of the outbreaks remained unknown at the time of reporting. The national housing order for poultry and captive birds in Denmark, implemented during the high-risk period, was lifted on 5 May 2026 (RTE, online). Mortality was observed in all outbreaks, with a high proportion of dead birds recorded in non-commercial establishments (range: 7–71%, median: 15%) compared to commercial establishments (range: 0–8%, median: 4%). Clinical signs were observed in 7 of the 9 outbreaks (78%), whereas no clinical signs were reported in the remaining 2 (22%). A total of 140 persons were reported as exposed.

*France*

During the current reporting period from 28 February to 4 June 2026, 6 HPAI A(H5N1) outbreaks in poultry were reported in France via ADIS. Additional data were collected and provided by the reporting country for 5 (83%) of these outbreaks (Annex B), whereas no additional information was available for the last one, as it occurred too close to the publication of this report. In total, 61,382 birds were affected across the 6 establishments. The 5 outbreaks for which additional data were available occurred between March and May in commercial establishments keeping turkeys for fattening (2/5, 40%), ducks for foie gras production (1/5, 20%), ducks for mixed purposes (breeding and foie gras production) (1/5, 20%), and mixed species for mixed purposes (ducks for foie gras production and broilers) (1/5, 20%). All ducks in the affected establishments were vaccinated. Mortality and clinical signs were reported in the 2 (40%) outbreaks involving turkeys for fattening, whereas this information was not available for the other 3 (60%) outbreaks involving vaccinated ducks and broilers. However, a very high number of dead birds was reported among broilers (270/300, 90%) in the mixed-species establishment. This establishment, as well as another establishment where vaccinated ducks were kept, were identified as part of passive surveillance, whereas the third establishment with vaccinated ducks was identified via active surveillance. The most likely source of introduction for all 5 outbreaks remained unknown at the time of reporting.

*Germany*

During the current reporting period from 28 February to 4 June 2026, 7 HPAI A(H5N1) outbreaks in poultry were reported in Germany via ADIS. Additional data were collected and provided by the reporting country for all these outbreaks (Annex B). A total of 84,992 birds were affected across the 7 establishments between February and April, including 6 (86%) commercial establishments and one (14%) non-commercial establishment. The outbreaks involved mixed species for mixed purposes (2/7, 28%), turkeys for fattening (2/7, 28%), broilers (1/7, 14%), mixed species for fattening (1/7, 14%), and turkeys for breeding (1/7, 14%). Mortality was reported in all outbreaks, although not always among all species present. High proportions of dead birds were recorded in one of the establishments keeping turkeys for fattening (2256/4699, 48%), the establishment keeping broilers (28/73, 38%), and the two mixed-species establishments for mixed purposes (74/198, 37%; 51/188, 27%). Clinical signs were reported in one outbreak (14%) involving turkeys for fattening, whereas this information was not available for the rest of the outbreaks (6/7, 86%). Outdoor access was available in the 3 (43%) establishments keeping mixed species, one of which was non-commercial, whereas no outdoor access was reported in the remaining 4 (57%) outbreaks. Indirect contact with wild birds was identified as the most likely source of introduction in all 7 outbreaks.

*Hungary*

During the current reporting period from 28 February to 4 June 2026, 3 HPAI A(H5N1) outbreaks in poultry were reported in Hungary via ADIS. Additional data were collected and provided by the reporting country for all these outbreaks (Annex B). In total, 13,910 birds were affected across the 3 establishments in early March, including ducks for breeding (1/3, 33.3%), ducks for foie gras production (1/3, 33.3%), and geese for foie gras production (1/3, 33.3%). All 3 establishments were commercial and did not provide outdoor access. In 2 outbreaks, indirect contact with poultry was identified as the most likely source of introduction, whereas in another outbreak, the source was indirect contact with wild birds. Mortality was reported in all 3 outbreaks, with a total of 333 dead birds recorded. A higher proportion of dead birds was reported in the geese (158/478, 33%). Clinical signs were observed in all 3 outbreaks, as well as decreased water consumption and neurological signs in one of the establishments (Nébih, online). A total of 32 persons were reported as exposed.

Apart from these HPAI A(H5N1) outbreaks, Hungary reported the detection of A(H9N2) clade G5.5 virus back in April in 7 establishments keeping chickens (broilers), all of which were located in a single geographical area within one settlement. Increased mortality in one establishment initially raised suspicions of HPAI, however, the birds tested negative for HPAI A(H5) and A(H7) viruses. Tests for infectious bronchitis virus returned positive and pathological examinations revealed tracheitis, presenting with a clinical picture typical of a co-infection (Belkasmi et al., 2020; Regragui et al., 2025). This prompted testing for A(H9N2) virus. As this specific clade had originally been restricted to countries outside the EU, such as those in the Middle East (Fusaro et al., 2024), poultry workers were interviewed about their travel history; however, no plausible link could be established. The source of introduction therefore remained unknown at the time of reporting. All affected establishments were stamped out, cleaned, and disinfected. After 2-3 weeks following repopulation, the birds will be tested again for A(H9N2) virus. Waterfowl flocks present in the affected county were tested for A(H9) virus prior to movement, but all these tests returned negative.

*Italy*

During the current reporting period from 28 February to 4 June 2026, 3 HPAI A(H5N1) outbreaks in poultry were reported in Italy via ADIS. No additional data to complement the information in ADIS were provided by the reporting country. A total of 2530 birds were affected across the 3 establishments, all of which were located in central Italy, outside the densely populated poultry areas in the north. These outbreaks included an establishment keeping pheasants and partridges for game purposes in the Umbria region, as well as two multi-species establishments in the Tuscany region. Epidemiological investigations revealed that 2 of the 3 establishments were linked by the introduction of asymptomatic pheasants from the Umbrian establishment to one of the multi-species establishments in the Tuscany region. Clinical signs were reported in all affected establishments, and a total of 228 (9%) dead birds were recorded.

*Montenegro*

During the current reporting period from 28 February to 4 June 2026, 4 HPAI A(H5N1) outbreaks in poultry were reported in Montenegro via ADIS. No additional data to complement the information in ADIS were provided by the reporting country. In total, 179 birds were affected. According to a national government report, the outbreaks occurred in non-commercial backyard establishments with outdoor access (Government of Montenegro, online). Mortality was reported in all establishments, with a total of 170 (95%) dead birds recorded. Clinical signs were reported in 3 of the 4 (75%) outbreaks.

*Netherlands*

During the current reporting period from 28 February to 4 June 2026, 7 HPAI A(H5N1) outbreaks in poultry were reported in the Netherlands via ADIS. Additional data were collected and provided by the reporting country for all these outbreaks (Annex B). In total, 312,598 birds were affected across the 7 establishments between March and May. The outbreaks occurred in commercial establishments without outdoor access, involving laying hens (4/7, 57%), broilers (2/7, 29%), and chickens for breeding (1/7, 14%). Mortality and clinical signs were reported in all 7 outbreaks, while the most likely source of introduction remained unknown at the time of reporting.

*Poland*

During the current reporting period from 28 February to 4 June 2026, 102 HPAI A(H5N1) outbreaks in poultry were reported in Poland via ADIS. Additional data were collected and provided by the reporting country for 97 (95%) of these outbreaks (Annex B), whereas no additional information was available for the last 5, as they occurred too close to the publication of this report. In total, 5,716,654 birds were affected across the 102 establishments between March and May, with most outbreaks occurring in the Mazowieckie, Wielkopolskie, and Warmińsko-Mazurskie regions (European Commission, online). All 97 outbreaks for which additional information was available occurred in commercial establishments keeping turkeys for fattening (29/97, 31%), laying hens (17/97, 18%), chickens for breeding (14/97, 14%), ducks for fattening (13/97, 13%), ducks for breeding (9/97, 9%), broilers (6/97, 6%), geese for fattening (3/97, 3%), chickens for mixed purposes (2/97, 2%), geese for breeding (2/97, 2%), and geese for mixed purposes (2/97, 2%). Outdoor access was reported in 7 of the 97 (7%) outbreaks, involving ducks for fattening (3/7, 43%), geese for mixed purposes (2/7, 29%), geese for breeding (1/7, 14%), and geese for fattening (1/7, 14%), whereas no outdoor access was available in the other 90 (93%) establishments. In 57 of the 97 (59%) outbreaks, indirect contact with wild birds was identified as the most likely source of introduction, whereas in another 20 (21%) outbreaks it was indirect contact with poultry, and in one case (1%), it was direct contact with wild birds. The most likely source of introduction remained unknown for another 19 (20%) outbreaks at the time of reporting. Mortality was reported in 92 of the 97 (95%) outbreaks, whereas no information was available for the remaining 5 (5%) outbreaks. The proportion of dead birds was generally low, with the exception of one establishment keeping geese for fattening (5577/5727, 97%) and one establishment keeping laying hens (26,100/29,100, 90%). Clinical signs were reported in 82 of the 97 (85%) outbreaks, whereas no clinical signs were observed in the remaining 15 (15%) establishments. A total of 615 persons were reported as exposed across 59 of the 97 outbreaks. On 28 May, Poland prohibited repopulation of establishments within restricted zones at a country level until further notice.

*United Kingdom (excluding Northern Ireland)*

During the current reporting period from 28 February to 4 June 2026, 4 HPAI A(H5N1) outbreaks in poultry were reported in the United Kingdom (excluding Northern Ireland) via WOAH-WAHIS. A total of 14,456 birds were affected. According to media reports and info notes sent from the reporting country to EC, 3 of the 4 (75%) outbreaks occurred in commercial establishments keeping ducks for breeding (n = 13,900), while one outbreak (25%) was reported in pheasants for breeding (n = 600) (BFRPA, online). The avian influenza prevention zone for poultry and captive birds in England was lifted on 4 June (GovUK, online-a).

*United Kingdom (Northern Ireland)*[9]

During the current reporting period from 28 February to 4 June 2026, one HPAI A(H5N1) outbreak in poultry was reported in the United Kingdom (Northern Ireland) via ADIS. Additional data were collected and provided by the reporting country for this outbreak (Annex B). The outbreak was reported on 11 March in a commercial establishment keeping mixed species (pheasants, chickens, geese) for mixed purposes (n = 1044), which had no outdoor access. Indirect contact with wild birds was identified as the most likely source of introduction. Mortality was observed, with a total of 25 (2%) dead birds recorded. No clinical signs were reported. A total of 31 persons were reported as exposed.

**Captive birds**

Between 28 February and 4 June 2026, 32 HPAI A(H5N1) outbreaks in captive birds were reported in 7 countries in Europe: Germany (13), Czechia (7), Poland (6), Denmark (2), France (2), Estonia (1), and Ukraine (1) (Table 1, Figure 2).

Most outbreaks occurred in non-commercial or backyard establishments keeping small numbers of mixed poultry species for private consumption, particularly in Germany, Czechia, Poland, and Denmark. Many of these flocks were composed of laying hens, ducks, geese, pheasants, and/or turkeys, which were often kept together. Mortality was reported in all outbreaks, and in some cases, sudden death was observed. Clinical signs were less frequently reported.

In addition, HPAI outbreaks continued to affect captive birds kept in zoological parks, such as in Germany, where a goose tested positive on 5 March 2026, and a black-necked swan was affected at Schwerin Zoo (NDR, online). At Stralsund Zoo, another HPAI outbreak at the beginning of March involved wild turkey, California quail, chicken, common merganser, and scarlet ibis. In France, HPAI was detected in ornamental swans and geese kept in a private park in Le Grand-Lucé (GPHIN, online-a).

**Wild birds**

During the current reporting period from 28 February to 4 June 2026, a total of 763 HPAI A(H5) virus detections in wild birds (with one HPAI virus detection potentially including more than one wild bird species) were reported in 27 countries in Europe: Germany (403), Denmark (66),

[9] In accordance with the Agreement on the Withdrawal of the United Kingdom from the EU, and in particular with the Windsor Framework, the EU requirements on data sampling are also applicable to the United Kingdom (Northern Ireland).

United Kingdom (excluding Northern Ireland) (62), Poland (51), Netherlands (35), Austria (30), Belgium (21), Sweden (21), Norway (17), Estonia (15), Finland (12), France (5), Czechia (3), Hungary (3), Romania (3), Iceland (2), Lithuania (2), Moldova (2), Spain (2), Bulgaria (1), Croatia (1), Greece (1), Ireland (1), Italy (1), Latvia (1), Portugal (1), and Slovenia (1).

Overall, 755 HPAI virus detections in wild birds were reported as A(H5N1), 5 as A(H5Nx), 2 as A(H5N5), and one was not further typed (Table 1, Figure 2). The overall number of HPAI virus detections reported in wild birds (763) in the current reporting period was 4 times lower than in the previous reporting period (2768) (Figure 3) but 3 times higher compared to the same period in the previous epidemiological year (277). Regarding the wild bird categories involved and considering that more than one wild bird species can be included in a single HPAI virus detection, HPAI A(H5) was mostly recorded in waterfowl (65%, 498/763), followed by raptors (19%, 141/763), colony-breeding seabirds (9%, 70/763), and 'other' wild bird species (6%, 48/763) (Figure 5).

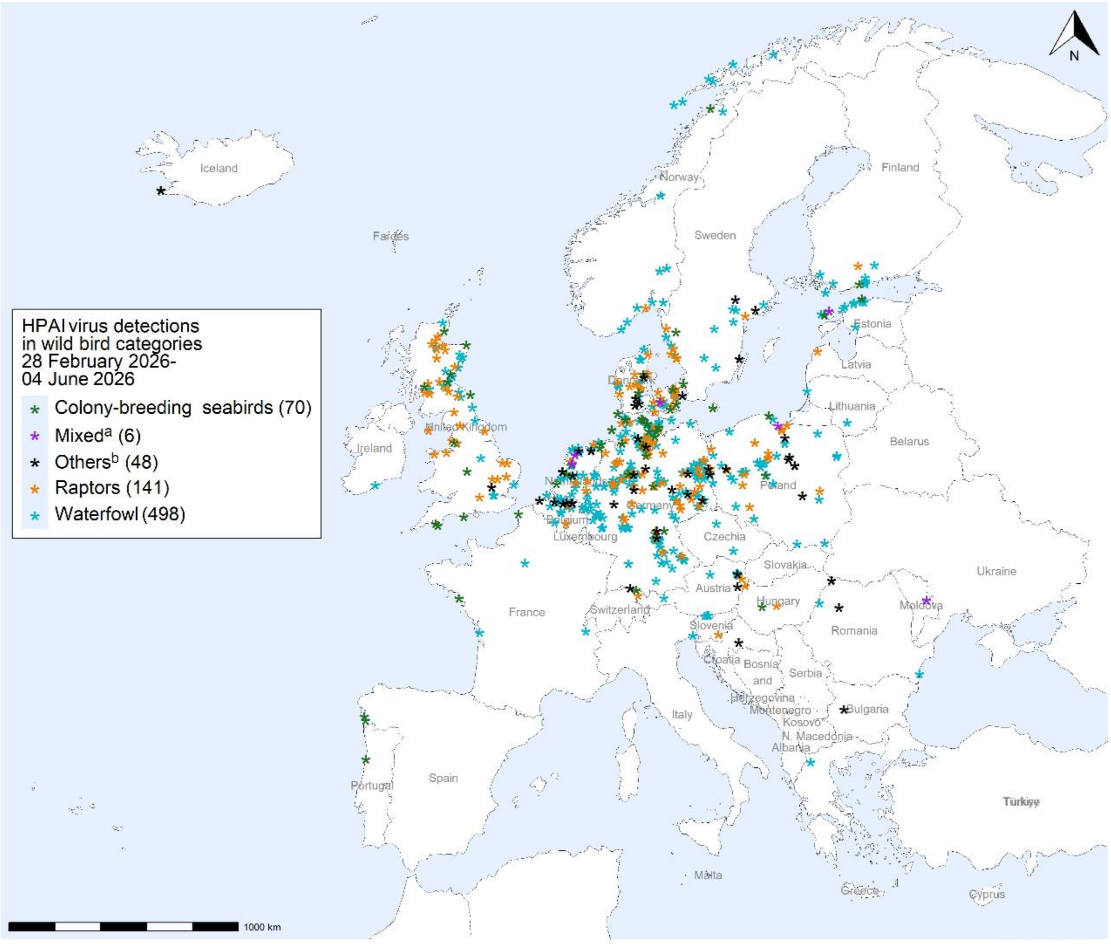

Author: EFSA
Data sources: ADIS, WOAH-WAHIS
Date updated: 04/06/2026

Figure 5: Geographic distribution, based on available geocoordinates, of HPAI virus detections in different categories of wild birds in Europe, by species category, from 28 February to 4 June 2026.

*Note*: the unit reported is the number of HPAI virus detections in different wild bird categories and not the total number of HPAI virus detections in wild birds (as more than one species can be involved in one single HPAI virus detection reported).

[a] Mixed, outbreaks in which multiple species or categories were involved.
[b] Others, all other affected categories which are not indicated in the legend.

* This designation is without prejudice to positions on status and is in line with United Nations Security Council Resolution 1244 and the International Court of Justice Opinion on the Kosovo Declaration of Independence.

*Source*: ADIS and WOAH-WAHIS (data extraction carried out on 4 June 2026).

The proportion of HPAI virus detections in waterfowl was slightly lower in the current (65%) compared to the previous reporting period (70%), while in raptors it was slightly higher (19% vs 14%). For colony-breeding seabirds (9% vs 8%) and 'other' wild bird species (6% vs 7%), the proportions were nearly the same. The geographical distributions of HPAI virus detections in the different wild bird categories largely overlapped (Figure 5).

The wild bird species in which HPAI viruses were detected belonged mainly to three orders: Anseriformes (509), Accipitriformes (125), and Charadriiformes (67). The main Anseriformes identified to species were the greylag goose (187 in the current vs 359 in the previous reporting period), mute swan (127 vs 515), and Canada goose (33 vs 183) (Figures A.1–A.2[10] in Annex A). In addition, there were 31 unidentified Anatidae. In the same period in the previous three years, considerably less Anseriformes, and mute swans in particular, were involved in the epidemic. Furthermore, only in 2021 were higher numbers of HPAI virus detections in greylag geese (190) and mute swans (156) reported in this period (EFSA, ECDC and EURL, 2021). Among the Accipitriformes identified to species, the Eurasian buzzard tested positive most frequently (81 vs 219), while there were 31 unidentified Accipitridae. The main Charadriiformes identified to species were the European herring gull (23 vs 57), black-headed gull (11 vs 45), and mew gull (5 vs 20). In addition, another 9 Laridae species remained unidentified. The complete list of wild bird species found as HPAI-affected from 28 February to 4 June 2026 is reported in Figure A.1 in Annex A. The number of HPAI virus-affected wild birds that were not identified to species was 93/780 (12%), around half the number recorded in the previous reporting period (600/2881, 21%) (Figures A.1–A.2 in Annex A).

Between 28 February and 4 June 2026, further sporadic detections of HPAI A(H5N5) virus were reported in wild birds, this time in a common raven and a black-legged kittiwake in Iceland.

The picture of HPAI-associated mortality of wild birds is incomplete, as reporting counts of dead wild birds in association with HPAI is not part of the current avian influenza surveillance system in Europe. Available data largely rely on voluntary efforts in some countries and media reports. Additionally, only a small proportion of wild birds found dead are submitted for HPAI testing. Therefore, HPAI virus detections in wild birds generally underestimate the number of wild birds actually dying from A(H5) virus infection.

#### 2.1.2 HPAI virus detections in birds outside Europe

An overview of HPAI virus detections in birds from countries outside Europe that were notified to WOAH from 28 February to 4 June 2026 is presented in Table 2 and Figure 6.

[10] Note that Figures A.1–A.2 in Annex A provide information on the numbers of wild bird orders/families/species that were detected as HPAI-affected at single bird level, as more than one bird can be involved in a single HPAI virus detection reported.

Table 2: Number of HPAI virus detections in non-European countries notified to WOAH, by virus subtype and country, from 28 February to 4 June 2026. Cumulative numbers since the start of the 2025–2026 epidemiological year are reported in parentheses (1 October 2025 to 4 June 2026).

| Region (total in season) | Country | Domestic birds | | | | Wild birds | | | | | Total |
|---|---|---|---|---|---|---|---|---|---|---|---|
| | | A(H5N1) | A(H5N9) | A(H5Nx) | A(H7N3) | A(H5N1) | A(H5N5) | A(H5N6) | A(H5N9) | A(H5Nx) | |
| Africa (44) | Côte d'Ivoire | 1 (1) | - | - | - | - | - | - | - | - | 1 (1) |
| | Namibia | - | - | - | - | 0 (1) | - | - | - | - | 0 (1) |
| | Nigeria | 2 (29) | - | - | - | - | - | - | - | - | 2 (29) |
| | Senegal | 1 (1) | - | - | - | - | - | - | - | - | 1 (1) |
| | South Africa | 0 (5) | - | - | - | 0 (7) | - | - | - | - | 0 (12) |
| Americas (710) | Argentina | 14 (20) | - | - | - | 2 (4) | - | - | - | - | 16 (24) |
| | Brazil | 1 (3) | - | - | - | 1 (1) | - | - | - | - | 2 (4) |
| | Canada | 8 (107) | - | - | - | 0 (1) | 0 (2) | - | - | - | 8 (110) |
| | Cayman Islands | 0 (1) | - | - | - | - | - | - | - | - | 0 (1) |
| | Chile | 26 (26) | - | - | - | 10 (10) | - | - | - | - | 36 (36) |
| | Colombia | 0 (8) | - | - | - | - | - | - | - | - | 0 (8) |
| | Guatemala | 0 (1) | - | - | - | - | - | - | - | - | 0 (1) |
| | Mexico | - | - | - | 1 (3) | 2 (5) | - | - | - | - | 3 (8) |
| | Peru | - | - | 25 (26) | - | - | - | - | - | - | 25 (26) |
| | US | 82 (452) | - | 0 (5) | - | 0 (4) | - | - | - | 0 (23) | 82 (484) |
| | Uruguay | - | - | - | - | - | - | - | - | 3 (8) | 3 (8) |
| Antarctica (1) | Heard Island and McDonald Islands | - | - | - | - | 0 (1) | - | - | - | - | 0 (1) |
| Asia (484) | Bhutan | 0 (3) | - | - | - | - | - | - | - | - | 0 (3) |
| | Cambodia | 3 (6) | - | - | - | - | - | - | - | - | 3 (6) |
| | China | - | - | - | - | 0 (1) | - | - | - | 0 (1) | 0 (2) |
| | India | 2 (20) | - | - | - | 0 (5) | - | - | - | - | 2 (25) |
| | Indonesia | 0 (4) | - | - | - | - | - | - | - | - | 0 (4) |
| | Iraq | 0 (4) | - | - | - | - | - | - | - | - | 0 (4) |
| | Israel | 0 (2) | - | - | - | 3 (12) | - | - | - | - | 3 (14) |
| | Japan | 4 (24) | - | - | - | 50 (137) | 0 (1) | - | - | 15 (26) | 69 (188) |
| | Mongolia | - | - | - | - | 0 (1) | - | - | - | - | 0 (1) |
| | Nepal | 51 (51) | - | - | - | 1 (1) | - | - | - | - | 52 (52) |
| | Philippines | 7 (32) | - | 0 (1) | - | - | - | - | - | - | 7 (33) |
| | Russia | - | - | - | - | 1 (2) | - | - | - | - | 1 (2) |
| | South Korea | 10 (56) | 1 (5) | - | - | 1 (52) | - | 0 (1) | 0 (10) | - | 12 (124) |
| | Taiwan | 10 (24) | - | - | - | 0 (2) | - | - | - | - | 10 (26) |
| | Viet Nam | 0 (2) | - | - | - | - | - | - | - | - | 0 (2) |
| **Total** | | **222 (882)** | **1 (5)** | **25 (32)** | **1 (3)** | **71 (247)** | **0 (3)** | **0 (1)** | **0 (10)** | **18 (58)** | **338 (1241)** |

–, no HPAI outbreaks were notified via WOAH-WAHIS.

*Source*: WOAH-WAHIS (data extraction carried out on 4 June 2026).

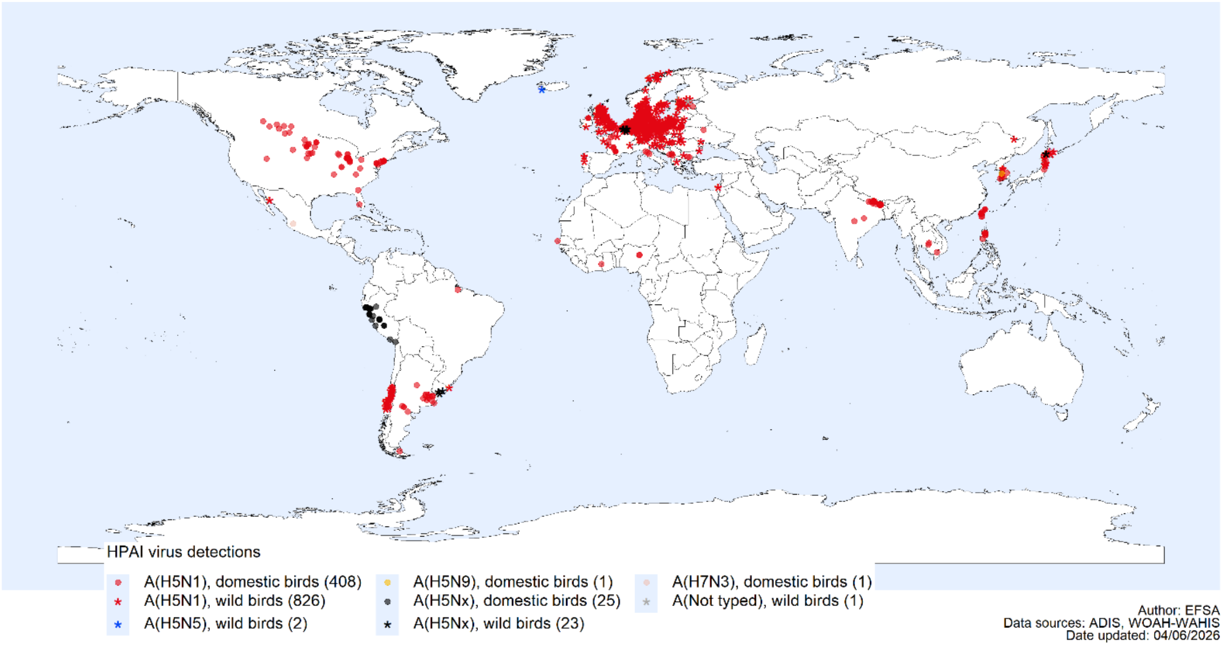

Figure 6: Geographic distribution, based on available geocoordinates, of HPAI virus detections reported worldwide in domestic (435) and wild (852) birds by virus type, from 28 February to 4 June 2026.

*Source*: ADIS and WOAH-WAHIS (data extraction carried out on 4 June 2026).

In comparison to the previous reporting period, the total number of HPAI virus detections in domestic and wild birds officially notified to WOAH from outside Europe decreased from 528 to 338, and the number of reporting countries outside Europe also decreased from 24 to 20 (Table 2, Figure 6). Compared to the same period in the previous year, the number of official notifications to WOAH during the current reporting period was slightly higher than the number of outbreaks reported between 28 February and 4 June 2025 (338 vs 293 outbreaks).

The tables and figures of the present report only include data extracted from WOAH-WAHIS on 4 June 2026. However, HPAI virus detections in domestic and wild birds are also reported to the public via different means. This additional information on HPAI virus detections available from sources other than WOAH-WAHIS has been integrated in the text below.

In Africa, the number of HPAI outbreaks officially notified to WOAH has decreased from 15 in the previous to 4 in the current reporting period. All 4 outbreaks were due to HPAI A(H5N1) viruses and reported from West Africa in domestic birds: (i) in a large poultry establishment in Côte d'Ivoire (95,000 birds); (ii) as described in the previous reporting period (Table A.1 in Annex A), the epidemic in poultry continued in Nigeria with two establishments affected this time (2400 and 10,000 birds in the two outbreaks, respectively); (iii) and in a small establishment in Senegal (311 birds). The Côte d'Ivoire event occurred after several years without major avian influenza reports in the country, indicating a reemergence of the virus in poultry systems (GPHIN, online-b). No wild bird outbreak was officially reported from Africa to WOAH in the current reporting period. A recent study however described the molecular characterisation of a common stern HPAI A(H5N1) virus from Namibia (2025–2026 epidemiological year), showing that it clustered within the EA-2024-DI.2 genotype and was closely related to contemporary European EA-2024-DI.2 viruses (Hamunyela et al., 2026).

As regards the Americas, the total number of HPAI virus detections in domestic and wild birds officially notified to WOAH from North America decreased from 225 to 82 for the US and from 28 to 8 for Canada, when comparing previous and current reporting periods (Table A.1 in Annex A). As before, Canada officially reported to WOAH only HPAI virus detections in domestic birds. The 8 outbreaks were reported in both backyard and larger establishments (26 to more than 33,000 birds). However, 9 HPAI A(H5N1) virus detections are reported on the website of the Canadian Food Inspection Agency: red-tailed hawk (3), Canada goose (2), American crow (2), barred owl (1), and peregrine falcon (1) (CFIA, online). The media also mentioned the detection of HPAI A(H5N1) virus in two common ravens in Yellowknife (CBC, online). In the US, 11 outbreaks in captive birds (flocks of 14 to 220 birds) and 71 outbreaks in poultry (wide range of establishment sizes) were officially notified to WOAH. The United States Department of Agriculture (USDA), in the same period, reported 104 outbreaks in all types of poultry establishments, mostly in turkeys and ducks for fattening (USDA, online-a), including also 26 smaller non-commercial or backyard establishments. The media highlighted that the commercial poultry sector was heavily impacted by HPAI A(H5N1), including very large flock losses (from tens of thousands up to over one million birds in single events) across states such as Arkansas (AOL, online), Iowa (WHO13, online), Massachusetts (MDAR, online), Michigan (WATTPoultry, online), Virginia (Fox news, online), and Wisconsin (24Tv, online). Furthermore, the USDA reported 582 HPAI virus detections in wild birds between 28 February and 4 June 2026 (USDA, online-b), substantially more than the detections officially notified to WOAH. Approximately 70 wild bird species were affected, most of which were Canada geese (25%, 134/582), red-tailed hawks (9 %, 54/582), and mallards (7%, 38/582) (USDA, online-b). The media also reported on these outbreaks in wild birds, mentioning large mortality events in different geese species (e.g. Canada geese) (Beacon, online-a) and vultures (GPHIN, online-c), with some local die-offs reaching hundreds of individuals. During the current reporting period, no new HPAI A(H5N5) virus detections were reported in the US or elsewhere outside Europe. In South America, as a continuation from the previous reporting period, Argentina and Brazil officially reported to WOAH 16 and 2 HPAI A(H5N1) virus detections, respectively. Argentina officially notified 13 HPAI outbreaks in captive birds (flocks of 13 to 336 birds), one outbreak in a 19,000-bird commercial poultry establishment, and 2 outbreaks in waterfowl (a coscoroba swan and a muscovy duck) in an urban ecological reserve in Buenos Aires (Canal26, online). In addition, the media further specified that the outbreaks in poultry in Argentina involved mainly chickens and ducks (Beacon, online-b). In Río Negro and Córdoba Provinces, affected backyard establishments kept various poultry species in addition to those, including geese and turkeys. One multi-species establishment in Río Negro Province showed clinical signs consistent with nervous, respiratory, and digestive involvement, suggesting intra-farm spread (Beacon, online-c). Brazil officially reported to WOAH an outbreak in captive birds (a flock of 90 birds), as well as an outbreak in coscoroba swans. The media also reported an outbreak at the Taim Ecological Reserve in Rio Grande do Sul, affecting mainly coscoroba swans (g1 Globo, online). Cases were concentrated around Lagoa da Mangueira, an important habitat for migratory bird species (ClicCamaqua, online). Chile officially reported to WOAH 26 HPAI A(H5N1) outbreaks in domestic birds and 10 HPAI A(H5N1) virus detections in wild birds (mostly coscoroba and black-necked swans), compared to none in the previous reporting period. The media focused on their impact on the country’s wild bird populations, particularly coscoroba and black-necked swans, across wetlands and protected areas from Valparaíso to Los Lagos (Biobio Chile, online). Additional affected wild birds included Fuegian steamer ducks, mallards, upland geese, and different raptor species (ADN Radio, online; Canal9, online; SAG, online-a). National surveillance identified 22 outbreaks affecting 739 wild birds across eight species in seven regions (CONAF, online). In poultry, multiple backyard establishments in several regions were involved, with chickens, ducks, geese, and turkeys affected among others (Beacon, online-d; SAG, online-b; SoyChile, online-a,b). Some outbreaks were characterised by high mortality and neurological signs (Catriel25, online). Commercial outbreaks affected laying hen establishments, including a large event in Talagante province, where approximately 700,000 birds were affected and increased mortality was reported (Publimicro, online). According to the media, also Colombia detected HPAI virus in backyard poultry, in the municipality of Puerto Concordia. Affected birds included chickens and other poultry species showing respiratory and neurological signs, with mortality reported. The outbreak was likely linked to contact with wild birds, as the affected establishment was located near wetland areas (Beacon, online-e). According to the Instituto Colombiano Agropecuario, this represented the first avian influenza outbreak detected in the country in 2026 (LAFM, online). In the current reporting period, Mexico officially reported to WOAH an HPAI A(H7N3) outbreak in a 7200-bird commercial poultry establishment and 2 HPAI A(H5N1) virus detection black vultures. Twenty-five outbreaks were officially notified to WOAH in captive birds (flocks of 7 to 602 birds) from Peru. These outbreaks were reported in backyard establishments across the Monsefú, Pomalca, Pucalá, and San José districts by the media and involved approximately 128 birds, all of which died and were confirmed HPAI A(H5N1)-positive by the national animal health authorities (Beacon, online-f). Furthermore, 3 HPAI virus detections in coscoroba swans were officially reported to WOAH from Uruguay. The media also reported avian influenza activity in wild birds, mainly affecting swans, including coscoroba swans, in wetland ecosystems (Subrayado, online; RadioCarve, online). Overall, while gulls were primarily affected during the early years of the HPAI A(H5N1) epidemic in the Americas, cases involving waterfowl species are now increasingly being reported.

No new HPAI outbreaks were reported from Antarctica between 28 February and 4 June 2026.

However, HPAI A(H5) outbreaks continued to be officially reported to WOAH from Asia (158 in total in the current reporting period) and, compared to the previous reporting period, an additional country was affected by HPAI (H5), namely Nepal (Table 2, Table A.1 in Annex A). In Cambodia, 2 captive bird flocks and one small-sized poultry establishment (10 to 147 birds) were affected. The media indicated that the backyard establishment housed chickens and ducks (Beacon, online-g). Exposure was also linked to a human who had direct contact with infected/dead chickens prior to testing positive. India officially notified to WOAH 2 HPAI A(H5N1) outbreaks in poultry (establishments with 2000 to 30,000 birds). In addition, the media reported the detection of HPAI A(H5N1) virus in wild birds in multiple species, including crows and peacocks (Jantaserishta, online-a; News18, online; The Kathmandu Post, online-a). At Guindy Children's Park, approximately 40 birds died or were affected, leading to temporary closure of the facility (The Hindu, online-a). In poultry, outbreaks affected chickens and mixed-species establishments in several states, including Chhattisgarh (Haberler, online, The Hindu, online-b), Kerala (Mathrubhimi, online), and Sikkim (Jantaserishta, online-b). Mortality and control measures involved thousands of birds, including reports of 22,000 chickens affected in Chhattisgarh (Haberler, online). Major control operations included culling of approximately 400,000 birds (The Indian Express, online-a), destruction of over 2 million eggs, and disposal of large quantities of poultry feed in Nandurbar district (Hindustan Times, online-a; The Indian Express, online-b). In the current reporting period, Israel officially notified to WOAH the detection of 3 HPAI A(H5N1) outbreaks in colony-breeding seabirds (2 Armenian gulls and one black-headed gull). Japan officially notified to WOAH 4 HPAI A(H5N1) outbreaks in large poultry establishments (8,000 to 460,000 birds), as well as 50 HPAI A(H5N1) and 15 A(H5Nx) virus detections in wild birds (63 large-billed crows, one carrion crow, one Japanese buzzard), respectively. According to the media, HPAI outbreaks reported in commercial chicken establishments mainly affected egg and meat production systems. Large-scale events included approximately 188,000 chickens in Hokkaido (China News, online-a), followed by a second major outbreak in Chitose City involving around 460,000 laying hens, and an additional outbreak detected in Tohoku Town, affecting approximately 230,000 chickens in a laying hen establishment (China News, online-b). In the current reporting period, Nepal officially reported to WOAH 51 HPAI A(H5N1) outbreaks in poultry establishments of various sizes (up to 227,000 birds) and onr HPAI A(H5N1) virus detection in a house crow. The media reported details on the commercial poultry outbreaks in Nepal across Jhapa, Morang, and Sunsari districts (Koshi Province). Large-scale impact included the culling of approximately 100,000 birds in those districts, with further reports indicating that 200,000 birds were targeted for depopulation in Koshi Province (GPHIN, online-d, The Kathmandu Post, online-b). In the Philippines, 7 HPAI A(H5N1) outbreaks were officially reported to WOAH in poultry flocks of 3 to 73,000 birds. At the same time, South Korea officially notified to WOAH 11 and one HPAI A(H5) outbreaks in domestic and wilds birds, respectively. For poultry, an HPAI A(H5N9) outbreak occurred in a 250,000-bird laying hen establishment (ChosunBiz, online), while A(H5N1) affected 10 medium-to-large poultry establishments (13,000 to 170,000 birds). According to the media, these commercial poultry establishments were located in multiple provinces (notably Chungcheong, Gyeonggi, and Jeolla) and kept chickens (laying hens, broilers, chickens for breeding) (Newsis, online-a,b; Yna, online), ducks for fattening (Newsis, online-c), and quails (CCtimes, online), with flock sizes ranging from 9,000 to 240,000 birds per outbreak. Extensive control measures were implemented, including large-scale culling, movement restrictions, quarantine zones, enhanced farm biosecurity, and surveillance in high-risk poultry operations (Newsis, online-c). The media also reported a higher number of outbreaks among wild birds this past winter than in previous seasons (HKBS, online). Taiwan officially reported to WOAH 10 HPAI A(H5N1) outbreaks in poultry establishments (6 to 80,000 birds). According to the media, these establishments were located in multiple counties (Changhua, Pingtung, Shuilin, Yunlin) and kept chickens (FTVNEWS, online; UDN, online), geese, and quails (CNA, online), with outbreaks detected both via passive and active surveillance. Reported flock sizes ranged from 883 geese to over 62,000 chickens and 78,000 quails, with cumulative depopulation events exceeding 24,000 chickens and nearly 80,000 quails in single operations. Finally, the media reported outbreaks in chickens in Bangladesh (Daily Amar, online), as well as in ducks and chickens in Viet Nam (NhanDan, online).

Shortly before the publication of this report, Australia reported the first detection of HPAI A(H5N1) virus in a brown skua on its western coast, which means that the virus is now present on every continent (BBC, online).

A list of all wild bird species that were reported to WOAH as HPAI-affected from outside Europe between 28 February and 4 June 2026 is presented in Table A.2 in Annex A.

#### 2.1.3 Genetic characteristics of HPAI viruses of the A(H5Nx) subtype in avian species

Details on the nomenclature of the HPAI A(H5) viruses used in this section are reported in Appendix B. Genotypes are assigned using the criteria described in Fusaro et al. (2024) and the tool GenIn2 (https://github.com/izsvenezie-virology/genin2). Mutation analyses of the HPAI A(H5N1) viruses have been performed using FluMut (Giussani et al., 2025) with the FluMutDB v6.4 mutation database (https://github.com/izsvenezie-virology/FluMutDB).

#### Genetic diversity of HPAI A(H5Nx) viruses in avian species in Europe

Since October 2025, more than 2300 complete genome sequences of European viruses from 29 countries have been characterised. All the viruses belong to clade 2.3.4.4b, which is the only clade detected in Europe since 2016.

Based on the genetic data available as of 19 June 2026, during the 2025–2026 epidemiological year, the vast majority (> 90%) of the sequenced viruses belonged to a single genotype, EA-2024-DI, sublineage EA-2024-DI.2.1. This genotype has been detected in wild (mainly Anseriformes) and domestic birds from all the 29 European countries for which data are available, representing the most widespread genotype in Europe, extending north from Iceland to Finland and south from Portugal to Bulgaria, reaching as far as Georgia. Outside Europe, the EA-2024-DI.2.1 genotype was identified in October 2025 in wild Anseriformes in Kazakhstan.

In addition to EA-2024-DI.2.1, since October 2025, A(H5N1) genotypes circulating during the previous epidemic waves, namely EA-2024-DI.2, EA-2024-DT, EA-2022-BB, EA-2023-DA, and the A(H5N5) genotype EA-2021-I, have occasionally been identified in few geographical areas. Genotype EA-2024-DI.2 has been detected in wild (mainly Anseriformes) and domestic birds in western Europe (United Kingdom, France, Spain, Portugal), as well as in a backyard flock in Austria. The viruses collected in western Europe cluster together with EA-2024-DI.2 viruses that had been circulating over the spring and summer months in these areas, suggesting a persistent viral circulation in the wild bird population. On the other hand, the virus collected from backyard birds in Austria cluster with viruses identified in eastern Europe during the 2024–2025 epidemiological year.

Genotypes EA-2024-DT and EA-2022-BB continue to circulate mainly among Laridae and other colony-breeding seabirds in separate geographical areas: EA-2024-DT in France, Spain, and Portugal; EA-2022-BB in Belgium, France, Norway, and the United Kingdom. Genotype EA-2023-DA continues to be detected exclusively in poultry in Bulgaria and Czechia; this genotype had not been detected in wild birds during the previous (2024–2025) and the current epidemiological year in other regions of Europe or outside Europe. The viruses collected in Bulgaria and Czechia cluster separately in the phylogenetic trees suggesting an ongoing separate circulation in an unknown ecological or domestic niche in the two countries. The A(H5N5) genotype EA-2021-I was detected at the end of January 2026 in a black-headed gull in Denmark.

In addition to the known genotypes, several reassortant viruses, in almost all cases originating from reassortment events involving mainly the PB2, PB1, or NP gene segments between the EA-2024-DI.2.1 and LPAI viruses have been characterised in Europe. All these new reassortants have been identified in one or very few outbreaks within one or two countries, indicating their scarce epidemiological relevance, except for two genotypes, namely EA-2026-FA and EA-2026-FB, which have been identified since January 2026 in wild and domestic birds in 5 and 3 countries, respectively. Specifically, genotype EA-2026-FA has been detected in eastern and central Europe (Romania, Germany, Czechia, Switzerland, Italy), while genotype EA-2026-FB circulated in northern-central Europe (Poland, Denmark, Sweden).

Based on the currently available data, the profile of circulating genotypes during the reporting period (March–May 2026) mirrors what has been observed throughout the entire epidemiological year, with no significant fluctuations in genotype frequency.

Since 28 February 2026, Poland reported 102 poultry outbreaks. The genetic data of about 40% of the viruses (sequences available for viruses collected up to April 2026) indicated the occurrence of multiple virus incursions from wild into domestic birds between February and March, followed by at least three separate inter-farm virus transmission events among premises located in the same geographic area.

**Mutations identified in HPAI A(H5Nx) viruses from avian species in Europe**

Molecular analyses of the HPAI A(H5N1) viruses circulating in birds in Europe since October 2024 indicate that these viruses do not contain critical mutations detected in previous pandemic strains in the receptor binding site of the HA protein, meaning that they continue to be well-adapted to avian species. Several mutations previously described in literature (Du et al., 2018; Suttie et al., 2019; Pinto et al., 2023) have been identified as being associated with i) enhanced polymerase activity and replication in mammals or mammalian cells, ii) increased virulence, iii) increased/conferred resistance towards antiviral drugs, iv) increased in vitro binding to human-type receptors α2,6-SA, v) decreased antiviral response in ferrets, vi) evasion of human butyrophilin subfamily 3 member A3 (BTN3A3), and vii) disruption of the second sialic acid (SIA) binding site in the neuraminidase protein, with a frequency varying by distinct mutations. The net effect of these mutations on the biological characteristics of the viruses is still unknown, and further research is needed to improve existing knowledge.

Similarly to the viruses collected during the previous epidemiological years (2020–2021, 2021–2022, 2022–2023, 2023-2024, 2024–2025), the circulating European viruses contain some of the mutations in the receptor binding domain of the HA protein that have been shown by in vitro assays studies to promote, to a certain extent, the recognition of human-type receptors of specific H5 viruses. In particular, some of them (i.e. S133A, S154N, T156A, H5 numbering) have been identified in the majority of the 2025–2026 A(H5N1) viruses, whereas others (i.e. D94N, G139R, HA1:S123P and HA2:R167K, T188I, N189D/T, P235S, E251K, S155N, Q192R, N193K, V210I) have only sporadically been observed (≤ 50 viruses). The impact of all these HA mutations on the biological characteristics of the circulating viruses is still unknown. Besides the mutations in the HA protein, it is important to mention that most of the A(H5N1) viruses belonging to the EA-2022-BB and EA-2023-DT genotypes contain mutations NP-Y52N and NA-A369I, which may increase their zoonotic potential due to the evasion of innate immunity and mammalian restriction factors or disruption of the second sialic acid binding site (2SBS).

Mutations associated with a reduced susceptibility of A(H5N1) viruses to the available antiviral drugs authorised for use in humans have rarely been identified in the circulating strains. Specifically, mutations associated with resistance to amantadine and rimantadine, NA, and PA inhibitors (WHO, online-a) have been detected in approximately 1.1%, 1%, and 0.2% of the analysed A(H5Nx) viruses collected in Europe since October 2025 (n > 2300), respectively. However, for PA inhibitors, it is important to mention that mutations have been studied in seasonal influenza viruses and not specifically in A(H5N1) viruses; the threshold of ≥ 3 $IC_{50}$ fold-change was used for the assignment of viruses with a reduced susceptibility to PA inhibitors.

Since October 2025, the K526R, E627K, M631L, and D701N mutations in the PB2 protein associated with virus adaptation in mammals (Suttie et al., 2019) have been detected in eleven European outbreaks in birds, more specifically in i) 10 A(H5N1) viruses collected from domestic, captive and wild birds from 7 different countries and ii) one A(H5N5) virus (genotype EA-2021-I) collected from a wild bird in Denmark. Based on the available data, a decreased frequency of detection of such mutations has been observed in birds, from about 4% in the 2024–2025 epidemiological year to about 0.5% in the 2025–2026 epidemiological year (based on data available up to 19 June 2026). Of note, all the A(H5N5) viruses of genotype EA-2021-I that have been collected in Europe since November 2024 contain the PB2 E627K/V molecular marker of mammalian adaptation, indicating that such mutation has become fixed in this virus population.

#### 2.1.4 Genetic characteristics of avian influenza viruses of other subtypes in avian species

Low pathogenic avian influenza A(H9N2) clade G5.5 (CDC, online-a) was identified in two genetically characterised samples collected from commercial poultry establishments in Hungary in April 2026. This clade is circulating in domestic birds in some countries in Africa, the Middle East and West Asia, and has previously never been detected in birds in the EU/EEA. Avian influenza A(H9N2) viruses belonging to clade G5.5 have also been reported in sporadic human cases, including Oman and Senegal in 2019, Ghana in 2024, and Italy in March 2026 in a patient who returned from West Africa (Pariani et al., 2026). The viruses that are most closely related to the Hungarian strains are A(H9N2) viruses of the same clade that had been circulating in the Middle East one or two decades ago. The long branches and time separating the Hungarian viruses from their progenitors suggest a significant gap of data, making it impossible to determine their origin.

Similarly to the majority of the A(H9N2) viruses of lineages G and B, they possess the HA-Q226L mutation (H3 numbering), which is associated with preferential binding to human-like α2-6-linked sialic acid (SA α2-6) receptors.

### 2.2 HPAI virus detections in non-human mammals

Between 28 February and 22 May 2026, HPAI A(H5) viruses were reported in wild, pet, and domestic mammals both in and outside Europe. Corresponding to the observed high circulation of HPAI viruses in wild birds during the current epidemiological year, the number of detections in mammals slightly increased. The data described in this report were actively collected from Member States and other European countries, retrieved via WOAH-WAHIS and from the USDA websites (for cases that occurred in the US), and supplemented with information from media reports (Table 3).

In Europe, HPAI A(H5N1) and A(H5N5) viruses were exclusively reported in wild mammals (total n = 31) in the current reporting period. A total of 16 dead red foxes were found as part of passive surveillance in Germany (10), Denmark (2), Sweden (2), Luxembourg (1), and the Netherlands (1); Belgium retrospectively reported 2 red foxes found dead at the end of 2025. Other positive detections included raccoons (7 in Germany), a European badger (Germany), a Eurasian otter (Sweden), a European polecat (Denmark), and a grey seal (Denmark), all of which were tested as part of passive surveillance. In Sweden, a European badger tested positive for HPAI virus after exhibiting neurological signs, including circling behaviour, and an impaired grey seal with difficulty breathing was found. All these mammals were positive for HPAI A(H5N1) virus. In contrast, Norway reported HPAI A(H5N5) virus detections in an 1-year-old polar bear and an adult walrus, which were found dead by tourist guides in northern Spitsbergen, on Svalbard. Two other polar bears in the same area reportedly showed clinical signs, including limping and possible paralysis of the hind legs (Veterinærinstituttet, online).

Following the serological detection of HPAI A(H5N1)-specific antibodies in one dairy cattle farm in the Netherlands, where cows were grazing outdoors back in October 2025 (EFSA, ECDC and EURL, 2026), further test results have now become available. Specifically, A(H5N1)-specific antibodies were detected in 20% (14/70) of the 70 individual milk samples collected from dairy cows at the same farm. Antibodies were moreover detected in 47% (34/72) and 63% (24/38) of the serum samples collected from lactating cows and young stock, respectively. No active infection or viral particles were found in the milk (RIVM, online). As a follow-up study, a total of 3,019 serum samples collected from cattle between November 2025 and January 2026 at 427 farms throughout the Netherlands were analysed. A higher proportion of samples came from Friesland, where antibodies had initially been detected. Of these, 30 (1%) samples tested positive in the initial NP-ELISA, but none tested positive after confirmatory testing using VNT or H5-specific ELISA (Ballmann et al., 2026). These results indicate non-specific or cross-reactions with other influenza viruses. Apart from the very low overall exposure of cattle to the virus throughout the country, the outcome of this study has therefore shown that the event in the Frisian farm can be considered an isolated case.

At the same time, several European countries with variable testing efforts actively reported the absence of HPAI viruses in mammals during the current reporting period: Finland, Italy, and United Kingdom (Northern Ireland)[11]. In addition, Austria shared detailed test results of analyses performed on samples from mammals (13 February to 22 May 2026), all of which resulted negative: pig (173), fox (17), horse (8), badger (1), bat (1), dog (1), and cattle (1) (personal communication by Sandra Revilla-Fernández, AGES). Italy shared the results of a retrospective study carried out on 3445 serum samples collected from dairy cows. The samples had been collected during the 2025–2026 epidemiological year in northern Italian provinces where HPAI A(H5N1) viruses were actively circulating in wild and domestic birds. All samples tested negative for A(H5) virus (personal communication by Calogero Terregino, AI-ND EURL). Up to 12 May 2026, Belgium tested another 78 mammals, all of which resulted negative: Canidae (63), Phocidae (11), Phocoenidae (3), and Mustelidae (1) (personal communication by Ingeborg Mertens, favv).

Outside Europe, no new HPAI virus detections were reported via WOAH-WAHIS in the current reporting period. However, according to the USDA (USDA, online-c), HPAI A(H5) virus was detected in the following wildlife species between 28 February and 22 May 2026: northern elephant seal (24), red fox (8), California sea lion (4), skunk (4), fox (2), grey seal (2), striped skunk (2), American mink (1), bobcat (1), and Virginia opossum (1). The mortality event in northern elephant seals, described in the previous reporting period, continued, with cases reported also in other marine mammal species. In total, 57 detections in northern elephant seals were reported between February 2026 and the end of the current reporting period (USDA, online-c). This was anticipated by detections in 4 sea otters and 3 California sea lions between November 2025 and February 2026 (USDA, online-c), all in California (CIDRAP, online-a). At Año Nuevo Natural Reserve, northern elephant seals exhibited abnormal respirations, tremors, and neurological signs, with infections first confirmed in dead pups at the beginning of the outbreak (CIDRAP, online-b). The USDA moreover retrospectively reported (February 2026) HPAI virus detections in skunk (2), striped skunk (1), and bobcat (1), as well as fisher (2025) and North American river otter (2024) (USDA, online-c). Up to 22 May 2026, the number of dairy cattle farms reportedly affected by the epidemic rose by 19 to 1,107 (with reinfections not counting towards that number) in 19 states (USDA, online-d). All new detections were in Idaho. As regards pets, two domestic cats in the US (New Jersey, Wisconsin) were reported (USDA, online-c). In Canada, a domestic cat in south-eastern Saskatchewan tested positive for HPAI A(H5N1) virus after developing sudden neurological and respiratory signs and dying. The animal

[11] In accordance with the Agreement on the Withdrawal of the United Kingdom from the EU, and in particular with the Windsor Framework, the EU requirements on data sampling are also applicable to the United Kingdom (Northern Ireland).

had outdoor access, suggesting potential exposure to infected wild birds or contaminated environments (Global news, online). A red fox is mentioned on the website of the Canadian Food Inspection Agency (CFIA, online).

Table 3: Avian influenza A(H5Nx) virus detections in mammalian species other than humans related to circulating viruses worldwide, 2016–2026.

| Virus | Animal (order) | Animal (family) | Animal (species) | Country | Reference[a] |
|---|---|---|---|---|---|
| A(H5N1) or A(H5Nx) clade 2.3.4.4b | Artiodactyla | Bovidae | Cattle (*Bos taurus*) | Netherlands[b], US | Ballmann et al. (2026) |
| | | | Goat (*Capra hircus*) | US | |
| | | | Sheep (*Ovis aries*) | Norway, UK | Fosse et al. (2025), GovUK (online-b,c) |
| | | Camelidae | Alpaca (*Lama pacos*) | US | |
| | | Suidae | Pig (domestic) (*Sus scrofa*) | Italy[b], US | Rosone et al. (2023) |
| | | | Pig (wild boar) (*Sus scrofa*) | Germany[b] | Günther et. al (2026) |
| | Carnivora | Canidae | Arctic fox (*Vulpes lagopus*) | Finland | |
| | | | Common raccoon dog (*Nyctereutes procyonoides*) | Finland, Germany[b], Japan, Sweden[b] | Günther et. al (2026) |
| | | | Coyote (*Canis latrans*) | US | |
| | | | Dog (*Canis lupus familiaris*) | Canada, Italy[b], Poland | Szaluś-Jordanow et al. (2024) |
| | | | Gray fox (*Urocyon cinereoargenteus*) | US | |
| | | | Island fox (*Urocyon littoralis*) | US | |
| | | | Japanese raccoon dog (*Nyctereutes viverrinus*) | Japan | |
| | | | Red fox (*Vulpes vulpes*) | Austria, Belgium, Canada, Denmark, Estonia, Finland, France, Germany[c], Ireland, Italy, Japan, Latvia, Luxembourg, Netherlands, Norway, Slovenia, Spain, Sweden[c], UK, US | Günther et. al (2026) |
| | | | South American bush dog (*Speothos venaticus venaticus*) | UK | |
| | | Felidae | Bobcat (*Lynx rufus*) | US | |
| | | | Canadian lynx (*Lynx canadensis*) | US | |
| | | | Caracal (*Caracal caracal*) | Poland, US | |
| | | | Cat (*Felis catus*) | Belgium, Canada, France[c], Germany, Hungary[c], Italy[b], Netherlands[c], Poland, South Korea, US | DutchNews (online), TF1 Info (online) |
| | | | Cheetah (*Acinonyx jubatus*) | US | |
| | | | Eurasian lynx (*Lynx lynx*) | Finland, Sweden[b], US | |
| | | | Leopard (*Panthera pardus*) | India, US, Viet Nam | The Times of India (online-a) |
| | | | Leopard cat (*Prionailurus bengalensis*) | South Korea | |
| | | | Lion (*Panthera leo*) | India, Peru, US, Viet Nam | Hindustan Times (online-b), The Times of India (online-b) |
| | | | Mountain lion (*Puma concolor*) | US | |
| | | | Serval (*Leptailurus serval*) | Bangladesh, US | |
| | | | Tiger (*Panthera tigris*) | India, US, Viet Nam | Hindustan Times (online-b), The Times of India (online-a,b) |

| Virus | Animal (order, family, species) | | Country | Reference[a] |
|---|---|---|---|---|
| | Mephitidae | Striped skunk (*Mephitis mephitis*) | Canada, US | |
| | Mustelidae | American marten (*Martes americana*) | US | |
| | | American mink (*Neovison vison*) | Canada, Finland, Spain, US | |
| | | Beech marten (*Martes foina*) | Netherlands | GISAID (online) |
| | | Eurasian otter (*Lutra lutra*) | Finland, France, Netherlands, Sweden, UK | Préfet des Landes (online) |
| | | European badger (*Meles meles)* | Germany[c], Netherlands, Sweden | Günther et. al (2026) |
| | | European pine marten (*Martes martes*) | Germany | |
| | | European polecat (*Mustela putorius*) | Belgium, Denmark, Netherlands | |
| | | Ferret (*Mustela furo*) | Belgium, Poland, Slovenia | Golke et al. (2024) |
| | | Fisher (*Pekania pennanti*) | US | |
| | | Long-tailed weasel (*Neogale frenata*) | US | |
| | | Marine otter (*Lontra felina*) | Chile | |
| | | North American river otter (*Lontra canadensis*) | US | |
| | | Sea otter (*Enhydra lutris*) | US | |
| | | Southern river otter (*Lontra provocax*) | Chile | |
| | | Stoat (*Mustela erminea*) | US | |
| | Odobenidae | Walrus (*Odobenus rosmarus*) | Norway | |
| | Otariidae | Antarctic fur seal (*Arctocephalus gazella*) | South Georgia and the South Sandwich Islands, Uruguay | Banyard et al. (2024), Bennison et al. (2024) |
| | | California sea lion (*Zalophus californianus*) | US | |
| | | Northern fur seal (*Callorhinus ursinus*) | Russia | |
| | | South American fur seal (*Arctocephalus australis*) | Argentina, Brazil, Peru, Uruguay | |
| | | South American sea lion (*Otaria flavescens*) | Argentina, Brazil, Chile, Peru, Uruguay | |
| | Phocidae | Caspian seal (*Pusa caspica*) | Russia | |
| | | Crabeater seal (*Lobodon carcinophaga*) | Joinville Island | Phys.org (online) |
| | | Grey seal (*Halichoerus grypus*) | Canada, Denmark, Germany, Netherlands, Poland, Sweden[c], UK, US | |
| | | Harbour seal (*Phoca vitulina*) | Canada, Denmark, Germany, Japan, Netherlands, South Georgia and the South Sandwich Islands, Sweden, UK, US | The Mainichi (online) |
| | | Northern elephant seal (*Mirounga angustirostris*) | US | |
| | | Southern elephant seal (*Mirounga leonina*) | Argentina, South Georgia and the South Sandwich Islands, Possession Island | Banyard et al. (2024), Bennison et al. (2024) PASTAAF (online-a,b) |
| | Procyonidae | Raccoon (*Procyon lotor*) | Canada, Germany[c], US | Günther et. al (2026) |

| Virus | Animal (order, family, species) | | | Country | Reference[a] |
|---|---|---|---|---|---|
| | | | South American coati (*Nasua nasua*) | Germany, Uruguay | |
| | | Ursidae | American black bear (*Ursus americanus*) | Canada, US | |
| | | | Asian black bear (*Ursus thibetanus*) | France | |
| | | | Brown bear (*Ursus arctos*) | US | |
| | | | Kodiak grizzly bear (*Ursus arctos horribilis*) | US | |
| | | | Polar bear (*Ursus maritimus*) | US | |
| | Cetacea | Delphinidae | Bottlenose dolphin (*Tursiops truncatus*) | Peru, US | |
| | | | Chilean dolphin (*Cephalorhynchus eutropia*) | Chile | |
| | | | Common dolphin (*Delphinus delphis*) | Peru, UK | Leguia et al. (2023) |
| | | | White-sided dolphin (*Lagenorhynchus acutus*) | Canada | |
| | | Phocoenidae | Burmeister's porpoise (*Phocoena spinipinnis*) | Chile | |
| | | | Harbour porpoise (*Phocoena phocoena*) | Sweden, UK | |
| | Didelphimorphia | Didelphidae | Virginia opossum (*Didelphis virginiana*) | US | |
| | Lagomorpha | Leporidae | Desert cottontail (*Sylvilagus audubonii*) | US | |
| | Rodentia | Cricetidae | Deer mouse (*Peromyscus* spp.) | US | |
| | | | Muskrat (*Ondatra zibethicus*) | US | |
| | | | Prairie vole (*Microtus ochrogaster*) | US | |
| | | Muridae | Black rat (*Rattus rattus*) | US | |
| | | | Brown rat (*Rattus norvegicus*) | Egypt, US | Kutkat et al. (2024) |
| | | | House mouse (*Mus musculus*) | US | |
| | | | House rat (*Rattus rattus*) | Egypt | Kutkat et al. (2024) |
| | | Sciuridae | Abert's squirrel (*Sciurus aberti*) | US | |
| | | | Eastern gray squirrel (*Sciurus carolinensis*) | US | |
| | | | Round-tailed ground squirrel (*Xerospermophilus tereticaudus*) | US | |
| | | | Thirteen-lined ground squirrel (*Ictidomys tridecemlineatus*) | US | |
| **A(H5N1) clade 2.3.2.1a** | Carnivora | Felidae | Cat (*Felis catus*) | India | Raut et al. (2025)<br>The Times of India (online-c) |
| **A(H5N5) clade 2.3.4.4b** | Carnivora | Canidae | Arctic fox (*Vulpes lagopus*) | Iceland, Norway | |
| | | | Red fox (*Vulpes vulpes*) | Canada, Norway | |
| | | Felidae | Bobcat (*Lynx rufus*) | Canada | |
| | | | Cat (*Felis catus*) | Canada, Iceland | |

| Virus | Animal (order, family, species) | | | Country | Reference[a] |
|---|---|---|---|---|---|
| | | | Eurasian lynx (*Lynx lynx*) | Norway | |
| | | Mephitidae | Striped skunk (*Mephitis mephitis*) | Canada | |
| | | Mustelidae | American mink (*Neovison vision*) | Iceland | |
| | | | Eurasian otter (*Lutra lutra*) | Norway | |
| | | | European pine marten (*Martes martes*) | Netherlands | |
| | | Odobenidae | Walrus (*Odobenus rosmarus*) | Norway | |
| | | Phocidae | Grey seal (*Halichoerus grypus*) | UK | |
| | | | Ringed seal (*Pusa hispida*) | Canada | |
| | | Procyonidae | Raccoon (*Procyon lotor*) | Canada | |
| | | Ursidae | Polar bear (*Ursus maritimus*) | Norway | |
| **A(H5N6) clade 2.3.4.4b** | Carnivora | Canidae | Dog (*Canis lupus familiaris*) | China | Yao et al. (2023) |
| | | Mustelidae | American mink (*Neovison vison*) | China | Zhao et al. (2024) |
| **A(H5N8) clade 2.3.4.4b** | Artiodactyla | Suidae | Pig (domestic) (*Sus scrofa*)[b] | France | Herve et al. (2021) |
| | | | Pig (wild boar) (*Sus scrofa*)[b] | Germany | Schülein et al. (2021) |
| | Carnivora | Canidae | Red fox (*Vulpes vulpes*) | UK | |
| | | Phocidae | Grey seal (*Halichoerus grypus*) | Poland, Sweden, UK | Shin et al. (2019), Floyd et al. (2021) |
| | | | Harbour seal (*Phoca vitulina*) | Denmark, Germany, UK | Floyd et al. (2021) Ärzteblatt (online), Avian Flu Diary (online), Outbreak News Today (online) |

[a] References other than those mentioned under source.

[b] Serological detection.

[c] Both virological and serological detection.

Source: EFSA, USDA (USDA, online-c,d) and WOAH-WAHIS (data extraction carried out on 4 June 2026).

#### 2.2.1 Genetic characteristics of HPAI viruses of the A(H5Nx) subtype circulating in Europe in non-human mammals

Details on the nomenclature of the HPAI A(H5) viruses used in this section are reported in Appendix B. Genotypes are assigned using the criteria described in Fusaro et al. (2024) and the tool GenIn2 (https://github.com/izsvenezie-virology/genin2). Mutation analyses of the A(H5N1) viruses have been performed using FluMut (Giussani et al., 2025) with FluMutDB v6.4 mutation database (https://github.com/izsvenezie-virology/FluMutDB).

Based on the genetic data available as of 19 June 2026, since October 2025 the complete or partial genome sequences of 27 HPAI A(H5N1) viruses of clade 2.3.4.4b collected from 6 distinct mammalian species (domestic cat, Eurasian otter, European badger, harbour seal, grey seal, red fox) in 9 different European countries have been analysed. Three viruses collected in March from a red fox, a Eurasian otter, and a grey seal in Sweden belong to the A(H5N1) genotype EA-2026-FB. This genotype was identified in multiple wild bird species in the country between January and March 2026. All the other characterised viruses from mammals belong to the A(H5N1) genotype EA-2024-DI.2.1.

Of note, 22/27 (81.5%) viruses contain the PB2 molecular marker of mammalian adaptation E627K, which has not been identified in the phylogenetically related viruses collected from avian species. Additionally, one virus acquired the PB2 substitutions E192K and Q591K (Yamada et al., 2010; Taft et al., 2015).

Additionally, two A(H5N5) viruses collected in May 2026 on Svalbard, Norway, from a walrus and a polar bear were characterised. Both viruses belong to genotype EA-2021-I and cluster with a group of A(H5N5) viruses of the same genotype collected between November 2024 and April 2025 in northern Europe and Canada. All these viruses share the mammalian adaptive marker PB2-E627V.

### 2.3 Avian influenza virus infections in humans

#### 2.3.1 Overview of the most recent human infections with avian influenza viruses

Since 28 February and as of 4 June 2026, 19 new human cases of infection with avian influenza viruses have been publicly reported from six countries and territories: Bangladesh (n = 2, one fatal), Cambodia (n = 3, one fatal), China (n = 12), India (n = 1), Italy (n = 1, imported case), and Taiwan (n = 1). These infections were caused by four different subtypes: A(H5N1), A(H5N6), A(H7N7), and A(H9N2) (Table 4). Most of the cases had exposure to poultry or live animal markets (Table 5). Sequences were available on GISAID's EpiFlu™ platform (Shu and McCauley, 2017) from one of the cases in Bangladesh, two of the cases in Cambodia, and the imported case from Italy. In addition, the sequence from a previously described case from China was also included and analysed (Table 6).

Table 4: Globally reported cases or detections of avian influenza virus in humans, including virus subtypes reported in the last 12 months.

| Subtype | Cases reported 28 Feb 2026–4 June 2026 | | | Cases reported since first report | | | |
|---|---|---|---|---|---|---|---|
| | Cases reported | Deaths | Reporting countries | First report | Cases reported | Deaths | Reporting countries |
| **H5N1** | 6 | 2 | 3 | 1997 | 1020[a] | 478[b] | 25 |
| **H5N2** | 0 | 0 | 0 | 2024 | 2 | 1 | 1 |
| **H5N5** | 0 | 0 | 0 | 2025 | 1 | 1 | 1 |
| **H5N6** | 1 | 1 | 1 | 2014 | 94 | 58[c] | 2 |
| **H7N7** | 1 | 0 | 1 | 2003 | 93 | 1 | 3 |
| **H9N2** | 11 | 0 | 2 | 1998 | 206 | 2 | 11 |
| **H10N3** | 0 | 0 | 0 | 2021 | 7 | 0 | 1 |

[a] Human case of A(H5) epidemiologically linked to A(H5N1) outbreaks in poultry and dairy cattle farms in the US are included in the reported number of A(H5N1) cases.
[b] Deaths among a total of 1000 cases reported 2003–4 June 2026. Mortality data are not available for cases reported prior to 2003.
[c] The number of deaths among A(H5N6) cases was updated by China through the International Health Regulations (2005) mechanism (WHO, online-a), resulting in an increase in the reported number of deaths compared with previous reports.

*Source*: line list maintained by ECDC.

Table 5: Identified exposure associated with human cases of avian influenza, reported globally 28 February–4 June 2026 by zoonotic influenza subtype.

| | Reported exposure[a] | Cases |
|---|---|---|
| **A(H5N1)** | Poultry | 5 |
| | Other | 1 |
| **H5N6** | Poultry | 1 |
| **H7N7** | Poultry | 1 |
| **A(H9N2)** | Poultry | 8 |
| | Other | 2 |
| | Exposure source unknown[b] | 1 |

[a] Exposure to more than one animal species can be reported for cases.
[b] No epidemiological exposure was identified after investigation.

*Source*: line list maintained by ECDC.

Table 6: Human strains of avian influenza included in phylogenetic and mutation analysis. Sequences include those deposited in GISAID's EpiFlu™ database (Shu and McCauley, 2017) from 28 February 2026 to 4 June 2026.

| Isolate name | Subtype | Clade | Collection date | Conti-nent | Country | Markers |
|---|---|---|---|---|---|---|
| A/Bangladesh/IEDCR-3260113035/2026 | A(H5N1) | 2.3.2.1a | 2026-01-29 | Asia | Bangladesh | e.g. A/Bangladesh/Khulna/IEDCR-icddr,b-IC2/2025 (EFSA, ECDC and EURL, 2025a) |
| A/Cambodia/26030075/2026 | A(H5N1) | 2.3.2.1e | 2026-03-27 | Asia | Cambodia | e.g. A/Cambodia/NIPH-25080754/2025 (EFSA, ECDC and EURL, 2025b) |
| A/Cambodia/SVH260173/2026 | A(H5N1) | 2.3.2.1e | 2026-04-17 | Asia | Cambodia | e.g. A/Cambodia/NIPH-25080754/2025 (EFSA, ECDC and EURL, 2025b) |
| A/Italy/1694/2026 | A(H9N2) | G5.5 | 2026-03-16 | Europe | Italy | NP:Y52N,HA:Q226L,NS1:D92E |
| A/Hunan/40087/2025 | A(H9N2) | B4.7.2 | 2025-09-28 | Asia | China | e.g. A/Hong Kong/VM24002346/2024(EFSA, ECDC and EURL, 2024) + MP:I27A(M2) |

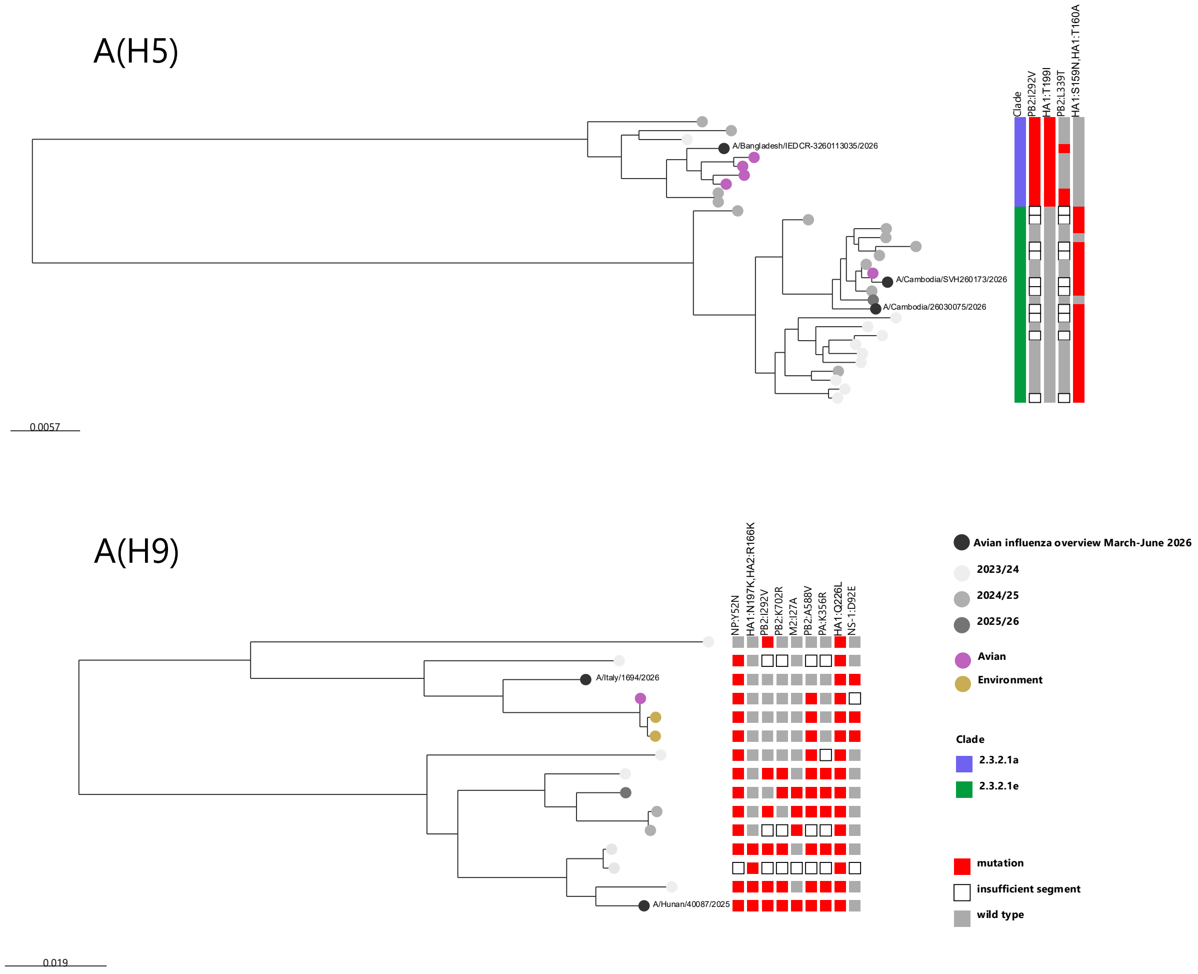

Figure 6: Phylogenetic tree of avian influenza virus strains from human cases, reconstructed from nucleotide alignment of the HA segment and annotated with significant mutation marker findings (H3 Numbering) across all segments. Strains from human cases described in previous reports since December 2023 are included and annotated according to epidemiological year. Closely related non-human strains are annotated according to their host.

#### 2.3.2 Human A(H5N1) cases

Between 28 February and 4 June 2026, six new human cases of influenza A(H5N1) virus infection, including two deaths, were publicly reported, from Bangladesh (2), Cambodia (3), and India (1). The cases were in four children and two adults.

**Bangladesh**

So far during 2026, two cases of influenza A(H5N1) infection, including one death, have been reported in Bangladesh. Both cases were in children from Chittagong and Sylhet divisions, with symptom onset in January and March 2026, respectively.

The first case was a child from Chittagong division, who developed symptoms (fever, acute respiratory symptoms, shortness of breath) on 21 January 2026, was hospitalised on 28 January, and died on 1 February (WHO, online-b). Exposure to live backyard poultry was reported prior to disease onset. Laboratory investigation revealed A(H5N1) virus belonging to clade 2.3.2.1a, similar to viruses that have been circulating in local poultry populations since around 2011 (WHO, online-b).

The case from Sylhet division developed symptoms on 27 March 2026, presenting with fever and cough, and was hospitalised on 28 March with a clinical diagnosis of measles and bronchopneumonia (WHO, online-c). As part of routine hospital-based influenza surveillance, a respiratory sample was collected on 29 March, which tested positive for influenza A(H5N1) by real-time reverse transcription polymerase chain reaction (RT-PCR) on 20 April. The patient was discharged on 30 March. No additional cases were identified among close contacts. Epidemiological investigations indicated exposure to household poultry.

The strain from the first case, A/Bangladesh/IEDCR-3260113035/2026, collected on 29 January 2026, was phylogenetically close to avian clade 2.3.2.1a strains collected in 2025 from the same country and had a mutation profile observed in previous cases from Bangladesh (Figure 6).

**Cambodia**

Between 28 February and 4 June 2026, Cambodia reported three new cases of human infection with avian influenza A(H5N1), including one death. The individuals were from Svay Rieng, Otdar Meanchey, and Banteay Meanchey provinces. Two were adults and one was a child. All three had exposure to sick or dead poultry in their households. No new cases have been reported among identified contacts of these cases.

The fatal case was an individual in their 60s from Svay Rieng province with underlying conditions, who developed symptoms (fever, shortness of breath) on 15 April 2026 after exposure to sick and dead household chickens. On 16 April, the patient was admitted to hospital, and a sample was collected on 17 April. Avian influenza A(H5N1) was confirmed by RT-PCR on 22 April. The patient died on the same day. No new cases were detected among 15 contacts of the case (WHO, online-c).

The other two cases, an asymptomatic adult woman from Banteay Meanchey province, from whom a sample was collected on 13 March, and a child < 5 years of age from Otdar Meanchey with symptom onset on 28 March, were identified during active case finding following the detection of A(H5N1) in sick and dead poultry (WHO, online-b). The child developed influenza-like illness symptoms and was hospitalised (WHO, online-b).

The two virus strains with sequence data available (one from the child and one from the fatal, adult case) cluster with other human and avian strains of clade 2.3.2.1e from Cambodia and contain the common marker S159N and T160A (H3 numbering) in HA1 (Figure 6), associated with increasing mammalian specificity of virus attachment to its haemagglutinin receptor (EFSA AHAW Panel and ECDC, 2025). No sequence data were available for segments PA, PB1 and PB2.

**India**

India reported its first human case of avian influenza A(H5N1) infection in 2026. A child from West Bengal state developed symptoms (fever and cough) on 19 March 2026, was admitted to hospital the same day, and discharged on 23 March. Genomic sequencing identified the A(H5N1)

virus as belonging to clade 2.3.2.1a, closely related to strains previously reported from Bangladesh and India in 2025 (WHO, online-c). No corresponding strains from India were available on GISAID EpiFlu™. The likely route of infection was through indirect exposure to poultry. No additional cases were reported among identified contacts (WHO, online-c).

Since the first report of human infection with avian influenza A(H5N1) virus in 1997, a total of 1020 cases have been reported to WHO by 25 countries (Figure 7). Since 2003, there have been 478 deaths out of the total 1000 cases reported.

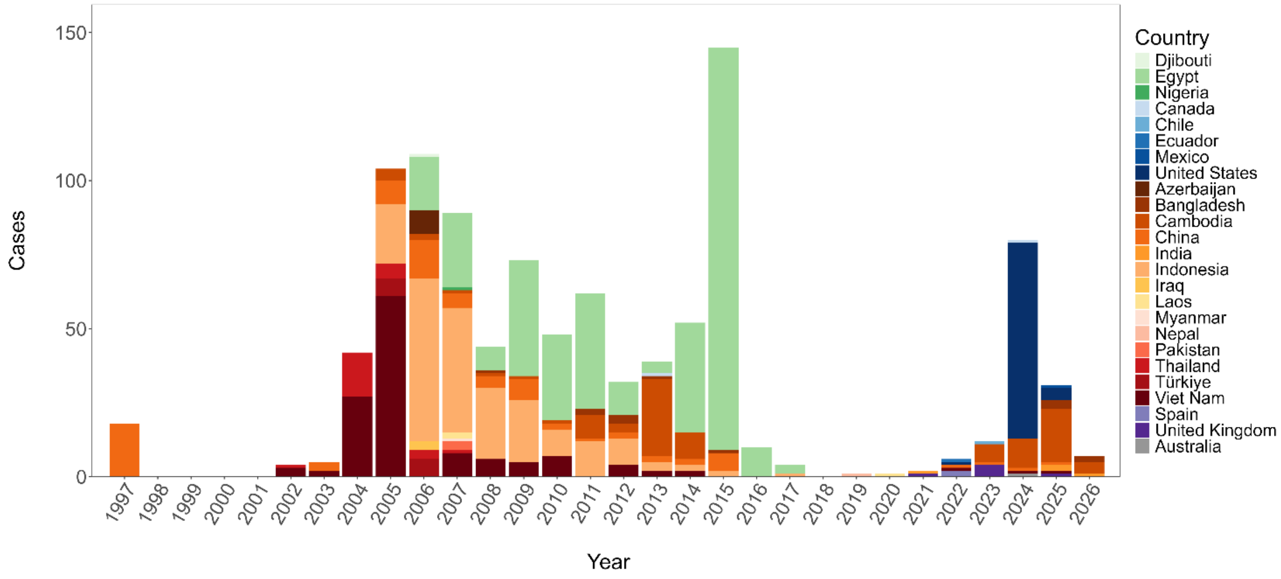

Figure 7: Distribution of reported human cases of A(H5N1) virus infection by year of onset or detection and reporting country from 1997 to 4 June 2026.

*Note*: The figure includes detections of A(H5N1) due to suspected environmental contamination reported in 2022 by Spain (2) and the US (1), and in 2023 by the United Kingdom (three detections, one inconclusive). Human cases of A(H5) epidemiologically linked to A(H5N1) outbreaks in poultry and dairy cattle farms in the US are included in the number of A(H5N1) cases.

*Source*: line list maintained by ECDC.

#### 2.3.3 Human A(H5N6) cases

In the reporting period from 28 February to 4 June 2026, one new fatal case of human infection with avian influenza A(H5N6) was reported in China (WHO, online-d). A woman in her 50s from Chongqing Municipality, China, developed symptoms on 16 April 2026. She was hospitalised on 23 April with severe pneumonia and died on 3 May. None of the case’s close contacts developed symptoms and all tested negative for influenza virus.

The patient had had exposure to live poultry, which was purchased, slaughtered, and consumed prior to symptom onset. Samples collected from the cutting board were positive for A(H5) virus.

The most recent, previous case of human infection with influenza A(H5N6) was reported in July 2024 in Anhui Province, China. Overall, since 2014, a total of 94 laboratory-confirmed human cases of avian influenza A(H5N6) (Figure 8), including 58 deaths (case fatality rate: 62%) (WHO, online-d), have been reported from China (93) and Laos (1) to WHO. Where known, the outcomes for A(H5N6) cases were updated by China through the International Health

Regulations (2005) mechanism, resulting in an increase in the reported number of deaths compared to previous reports in 2024. The majority of cases (> 90%) reported exposure to domestic poultry.

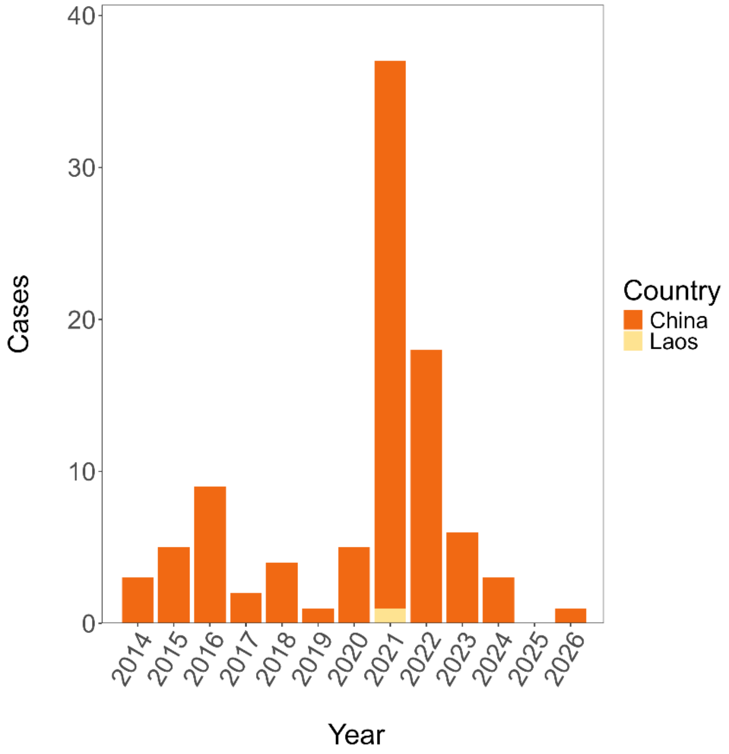

Figure 8: Distribution of reported human cases of A(H5N6) virus infection by year of onset or detection and reporting country from 2014 to 4 June 2026.

#### 2.3.4 Human A(H7N7) cases

Between 28 February and 4 June 2026, one human infection with avian influenza A(H7N7) was reported in Taiwan (Taiwan CDC, online-a). This is the first human case of A(H7N7) reported in Taiwan and the first case reported globally since 2013. The infected individual was a poultry farmer in their 70s, with underlying conditions, who developed symptoms (rhinorrhoea, myalgia, cough) on 20 March 2026. The individual was admitted to hospital with pneumonia on 22 March, received antiviral treatment and, following clinical improvement, was discharged on 3 April 2026. The patient had had exposure to domestic poultry prior to the onset of symptoms.

Low pathogenic avian influenza A(H7N7) was confirmed through sequencing from sputum samples collected on 27 March 2026. No mutations suggesting enhanced avian-to-human transmission or drug resistance were detected. Genomic analysis showed that the H7 belonged to the Eurasian lineage, similar to those strains circulating in Taiwan in wild birds (mainly geese and ducks) (Taiwan CDC, online a-b).

Contact tracing identified 33 contacts of the case who were monitored, including three who received prophylactic treatment; tests taken from six family members were negative for avian influenza. Environmental samples from the affected farm tested negative for avian influenza virus. The health and agricultural authorities have strengthened the monitoring of humans and animals, including monitoring for respiratory viruses and pneumonia at medical institutions and active monitoring of poultry farms and migratory birds. The authorities are cooperating with farmers to promote personal protective measures and public health education for livestock operators (Taiwan CDC, online-b).

In the EU/EEA, 92 human cases with A(H7N7) infection have been reported: 89 in the Netherlands in 2003 and three in Italy in 2013. For both events, most patients had had occupational exposure to poultry. Most cases had mild symptoms, including influenza-like symptoms and/or conjunctivitis. One fatal case was reported in a patient with pneumonia in combination with acute respiratory distress syndrome (EFSA AHAW Panel and ECDC, 2025). Since 2013, no further cases have been reported in the EU/EEA. Genetic analysis of the recent case in Taiwan showed that the virus was substantially different from the A(H7N7) viruses associated with human infections reported in Europe in 2003 and 2013 (Taiwan CDC, online-a).

#### 2.3.5 Human A(H9N2) cases

Between 28 February and 4 June 2026, 11 new human cases of influenza A(H9N2) virus infection were reported from China (10) and Italy (imported from Senegal). Among the reported cases, three were adults and eight were children; four developed pneumonia, and no deaths were reported. Eight patients were admitted to hospital. Eight of the cases had had exposure to live poultry, either at poultry markets or in a backyard setting, and two cases had possible indirect exposure to poultry. Cases had onset of symptoms between January and May 2026. In China, cases were reported from Guangxi, Yunnan, Sichuan, Jiangxi, and Guangdong provinces. No new cases have been detected among contacts of the affected individuals (WHO, online-b,c,e,f,g). Sequence data from a case in China described in the previous report (EFSA, ECDC and EURL, 2026) were available. The strain, A/Hunan/40087/2025 of clade B4.7.2 (WHO, 2026), was phylogenetically similar to a strain from Hong Kong (A/Hong Kong/VM24002346/2024) described in a previous report (EFSA, ECDC and EURL, 2024). A/Hunan/40087/2025 shares the mutation profile of A/Hong Kong/VM24002346/2024, with the addition of I27A in M2, observed in other related strains from human cases but very uncommon in avian strains and associated with increased resistance to amantadine (Suttie et al., 2019).

For the first time, a human case of avian influenza A(H9N2) infection has been reported in Europe, in the Lombardy region of Italy in a traveller returning from Senegal, where avian influenza A(H9N2) virus has previously been identified in birds. The patient had co-existing medical conditions and was hospitalised. Contact tracing was performed among passengers on the return flight from Senegal and contacts in Italy. Of 13 contacts identified, eight were tested for influenza virus and all were negative. No direct contact with animals was identified during the epidemiological investigation. (ECDC, 2026; Pariani et al., 2026). The strain, A/Italy/1694/2026, falls into a branch of clade G5.5 together with avian and environmental strains from Senegal collected in 2023, where the notable markers HA1:Q226L, NP: Y52N and NS1:D92E, detected in A/Italy/1694/2026, were also present.

Since the first report of human infection with avian influenza A(H9N2) virus in 1998, a total of 206 cases, including two deaths, have been reported to WHO (Figure 9).

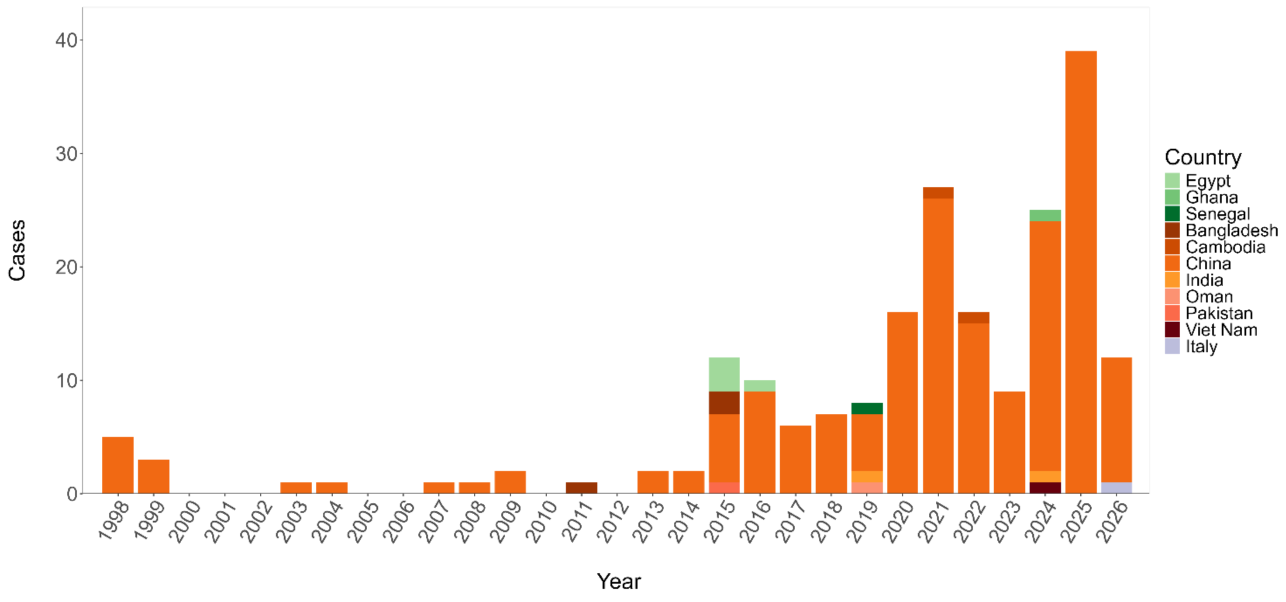

Figure 9: Distribution of reported human cases of A(H9N2) virus infection by year of onset or detection and reporting country, from 1998 to 4 June 2026.

*Source*: line list maintained by ECDC.

#### 2.3.6 Additional information relevant for public health and international risk assessments

In two recent human cases, the avian influenza A(H9N2) case reported in Italy in March 2026 (Pariani et al., 2026) and the avian influenza A(H5N5) case in the US in October 2025 (Lite et al., 2026), nasal or nasopharyngeal swabs were initially negative for influenza A virus. The A(H9N2) case was found to be positive for influenza A from testing of a bronchoalveolar lavage specimen two days after admission, while the A(H5N5) case was identified as positive for influenza A on Day 15 from a nasal swab (Ct value 34.6) and bronchoalveolar lavage specimen (Ct value 23.8). In order to avoid delays in diagnosis, collection and testing of alternative specimens, such as sputum or bronchoalveolar lavage, should be considered in severe cases where there is a suspicion of infection with zoonotic influenza virus.

Between November 2024 and January 2025 in Los Angeles County, US, 139 persons were exposed to cats with suspected or confirmed influenza A(H5N1) infection. Of the 139 exposed individuals, 30 experienced symptoms of influenza-like illness, but none of the tested individuals were positive for influenza A(H5) using RT-PCR. All those exposed were offered serological testing for influenza A(H5N1), including asymptomatic individuals, and 25 people from veterinary practices agreed to participate. Antibodies against influenza A(H5N1) clade 2.3.4.4b were detected in an asymptomatic individual, suggesting previous exposure to the virus and potential cat-to-human transmission (Vaughan et al., 2026). In geographical areas where susceptible pets may be exposed to birds infected with avian influenza, animal health professionals and pet owners should be made aware of the risk of infection in pets and of personal protective measures to reduce the risk of exposure when handling potentially infected animals.

Candidate vaccine viruses for zoonotic influenza viruses were reviewed at the WHO vaccine composition meeting in February 2026 based on recently characterised zoonotic influenza viruses to enhance pandemic preparedness. Based on the currently available epidemiological, genetic, and antigenic data for avian influenza A(H5), A(H7), and A(H10N3) viruses detected in animals and humans, no new candidate vaccine viruses were proposed. For influenza A(H9N2), a new candidate vaccine virus, A/Hunan/40087/2025-like, belonging to clade B4.7.2, was proposed (WHO, 2026).

The Food and Agriculture Organization of the United Nations (FAO), WHO and WOAH tripartite assessment of the global public health risk posed by HPAI A(H5) viruses remains unchanged in the most recent update on 18 May 2026. The risk is considered low for the general public and low-to-moderate for occupationally or frequently exposed individuals (FAO, WHO and WOAH, online-a). In the latest summary and risk assessment of 'Influenza at the human-animal interface' (WHO, online-h), WHO's assessment of the risk to public health for the currently known avian influenza viruses has not changed and is still considered to be low.

During the reporting period, the US Centers for Disease Control and Prevention (CDC) assessment of the public health risk posed by avian influenza A(H5) viruses has not changed and is currently considered low in the US (CDC, online-b).

Similarly, during the reporting period, the United Kingdom Health Security Agency (UK HSA) risk assessment of HPAI A(H5) to the general public, as available in 'Bird flu (avian influenza): latest situation in England' updated on 4 June 2026, has remained unchanged and is still considered to be very low (GovUK, online-a).

#### 2.3.7 ECDC risk assessment

ECDC's assessment of the risk of human infection with HPAI A(H5N1) clade 2.3.4.4b viruses remains unchanged. Overall, ECDC assesses the risk posed by HPAI A(H5N1) clade 2.3.4.4b viruses currently circulating in animals in Europe to be low for the general public in the EU/EEA, and low-to-moderate for those occupationally or otherwise exposed to infected animals or contaminated environments. The assessment considers the risk of infection at population level in the EU/EEA, taking into account the likelihood of human infection and transmission and the potential impact of such an infection, based on ECDC's framework for risk assessments (ECDC, 2019).

The risk assessment was informed by available evidence related to the transmission, prevalence, and characteristics of A(H5N1) viruses currently circulating in animals in Europe, as well as knowledge acquired from human cases of influenza A(H5N1) reported globally. This evidence is set out below.

The likelihood of human infection with HPAI A(H5N1) clade 2.3.4.4b viruses in the EU/EEA:

- Despite the extensive circulation of avian influenza A(H5N1) virus in animal populations in Europe over the past few years, with frequent opportunities for human exposure, there have been no confirmed cases of A(H5N1) clade 2.3.4.4b infection in humans in the EU/EEA, and transmission from infected animals to humans remains a rare event globally.
- In Europe, mutations associated with viral adaptation to mammalian hosts have been identified in sequences of A(H5N1) clade 2.3.4.4b viruses from mammals and birds. However, circulating viruses retain a binding preference for α2–3 sialic acid (avian-type) receptors and are still considered to be avian-like. There is currently no evidence of the viruses being more capable of infecting humans or having the ability to transmit between humans. Further understanding of the implications of such mutations for mammalian adaptation, infection and transmission will help in assessing any change in the associated risk to humans.
- Sporadic cases of infection with avian influenza A(H5N1) virus have been reported in humans globally, often with a history of unprotected exposure to poultry, cattle, or contaminated environments. Of the six human cases of influenza A(H5N1) publicly reported worldwide between 28 February and 4 June 2026, five had documented exposure to poultry and one had probable indirect exposure to poultry.
- To date, there has been no evidence of human-to-human transmission of influenza A(H5N1) clade 2.3.4.4b viruses.

The impact of human infection with HPAI A(H5N1) clade 2.3.4.4b viruses:

- Clinical presentations of individuals infected with A(H5N1), including clades other than 2.3.4.4b, have ranged from asymptomatic or mild (such as conjunctivitis and upper respiratory tract symptoms) to severe illness resulting in death, with a case fatality estimated at 48% since 2003. However, this figure is only based on reported cases and may be overestimated.
- The majority of human cases of infection with influenza A(H5N1) clade 2.3.4.4b reported in the US between April 2024–February 2025, as well as the human case reported in the United Kingdom in January 2025, experienced mild symptoms, such as conjunctivitis or mild respiratory illness. Between 28 February and 4 June 2026, no new cases of human infection with an A(H5N1) clade 2.3.4.4b virus were reported globally. The most recent reported case of human infection with influenza A(H5N1) characterised as clade 2.3.4.4b was reported by Mexico in April 2025 (WHO, online-i).
- The reasons for the variation in outcome following infection with influenza A(H5N1) virus are likely to be multi-factorial and may be attributed to the virus genotype, duration of exposure, viral load, transmission route, individual health status, personal protective measures taken, and medical treatment provided (FAO, WHO and WOAH, online-b). The detection of several mild cases of A(H5N1) virus infection and seropositive individuals without a history of clinical symptoms in the US suggests that mild and asymptomatic cases may be more common than previously reported for A(H5N1). Targeted surveillance of individuals exposed to infected animals is therefore recommended (EFSA AHAW Panel and ECDC, 2025).
- Most of the circulating A(H5N1) clade 2.3.4.4b viruses detected in Europe in the epidemiological year 2025–2026 remain susceptible to antiviral medicines available to treat humans, including adamantanes, neuraminidase inhibitors (e.g. oseltamivir) and polymerase acidic inhibitors (e.g. baloxavir marboxil).

Sporadic human infections with avian influenza viruses are likely to continue occurring in areas where the virus is prevalent and individuals have unprotected contact with infected animals or their contaminated environments. Circumstances or conditions that increase mammalian or human exposure to avian influenza viruses may lead to spillover events and further transmission, increasing the probability of avian influenza viruses adapting to mammals (EFSA AHAW Panel and ECDC, 2025). Given the ongoing transmission of A(H5N1) in wild birds and poultry, and in some mammals in certain settings, viral evolution through mutations or reassortment may occur, which could change the current assessment. Therefore, ECDC is regularly reviewing the risk assessment, taking into consideration any new developments or information that becomes available.

## 3 Summary and conclusions

### 3.1 Birds

- As in previous years, in line with the seasonal dynamics of avian influenza, the number of HPAI virus detections in wild birds has declined to a low level by the end of the current reporting period. This suggests that in the coming months, the risk of infection for breeding wild birds and their offspring, as well as the risk of spillover to other host groups—poultry, captive birds, wild and domestic mammals—will also decline. Nevertheless, sporadic outbreaks may occur over the coming months.
- Overall, the number of HPAI virus detections in wild birds in the current reporting period and in the current epidemiological year in general was substantially higher than in the same periods in the previous four years (2022 to 2025). However, no mass mortality of certain wild bird species was observed this spring.
- Waterfowl accounted for the majority of HPAI outbreaks in wild birds, while only low numbers of colony-breeding seabirds were affected. Those included several species of geese, with greylag geese being the waterfowl species with by far the highest number of HPAI virus detections in the current reporting period, at a level comparable to that in the same reporting period in 2021. Compared to the previous reporting period, there was a lower but still substantial number of Eurasian buzzards involved between March and May 2026.
- In the current reporting period, HPAI outbreaks in poultry in Europe occurred primarily in Poland, probably due to a contribution of multiple primary introductions as well as secondary spread. In the other countries, almost all HPAI outbreaks in poultry were associated with indirect or direct contact with wild birds.
- France continued to report sporadic HPAI outbreaks in vaccinated ducks, although to a lesser extent than in the previous two reporting periods.
- Chickens, particularly laying hens and breeders, were the most affected poultry species in Europe in the current reporting period.
- HPAI outbreaks in captive birds in Europe once again involved a large proportion of non-commercial or backyard establishments as well as zoo birds.
- After a short period with no reports, HPAI A(H5N5) virus was once again detected in two wild birds in Iceland.
- All HPAI A(H5N1) viruses identified in Europe since October 2025 belong to clade 2.3.4.4b and most of them to the genotype EA-2024.DI.2.1. This genotype has been identified all over Europe. The frequency of detection of PB2 mammalian adaptive mutations decreased in birds, from about 4% in the 2024–2025 epidemiological year to about 0.4% in the 2025–2026 epidemiological year.
- LPAI A(H9N2) virus of clade G5.5 was reported for the first time in seven broiler establishments in a single settlement in Hungary. Co-infection between this virus and infectious bronchitis virus was reported, which resulted in clinical signs and increased mortality. This specific clade of LPAI A(H9N2) virus is endemically circulating in domestic birds in some countries in Africa, the Middle East, and West Asia, and has never been detected in birds in Europe. Similarly to the majority of the A(H9N2) viruses of clade G5.5, the viruses identified in Hungary possess the HA-Q226L mutation (H3 numbering) associated with preferential binding to human-like α2-6-linked sialic acid (SA α2-6) receptors.
- Compared to previous years, more outbreaks were officially notified to WOAH from South America, affecting both domestic (commercial and non-commercial) and wild birds.

Among the most frequently affected species, there has been a shift from colony-breeding seabirds to waterfowl, with mass mortality of coscoroba swans reported from several countries.

- In general, and as previously reported, low numbers of wild birds infected with HPAI viruses were reported from outside Europe via official notifications to WOAH. Despite these low numbers, HPAI viruses were increasingly reported in geese from North America and crows from several Asian countries.
- In the current reporting period, Nepal reported a substantial number of HPAI outbreaks in poultry.
- In addition to HPAI A(H5N1) virus, HPAI A(H5N9) was reported in laying hens in South Korea, and HPAI (H7N3) was reported in domestic birds in Mexico.
- Shortly before the publication of this report, Australia reported the first detection of HPAI A(H5N1) virus on its mainland, which means that the virus is now present on every continent.

### 3.2 Mammals

- The number of HPAI virus detections in wild carnivores in Europe remained at a similarly low level compared to the previous reporting period. Red foxes were the mammal species in which HPAI A(H5N1) virus was most frequently identified.
- Although only found in two wild birds in the current reporting period, HPAI A(H5N5) virus was detected in a dead polar bear and a dead walrus on Svalbard, Norway, where two additional polar bears exhibited clinical signs.
- In the Netherlands, follow-up investigations after the detection of HPAI A(H5N1)-specific antibodies in dairy cattle back in January 2026 serologically confirmed the spillover of HPAI A(H5N1) virus to the dairy farm. A nationwide retrospective survey conducted among dairy farms suggested that this was an isolated event.
- Outside Europe, HPAI virus detections in mammals were only reported from the US and Canada, affecting predominantly wild carnivores and marine mammals. Mortality events associated with HPAI A(H5N1) virus in northern elephant seals were reported from various locations along the California coast, along with mortality of California sea lions and sea otters, which were affected by the virus for the first time.
- New HPAI virus detections in dairy cattle in the US were reported, confirming ongoing circulation of the virus in this population.
- Genetic data available for 29 A(H5Nx) viruses collected from wild and domestic mammals in Europe during the current epidemiological year indicate that they belong to clade 2.3.4.4b, A(H5N1) genotypes EA-2024-DI.2.1 (83%) and EA-2026-FB (10%), as well as A(H5N5) genotype EA-2021-I (7%). Of those, 86% are distinguishable from genetically related viruses identified in avian species by the presence of molecular markers of adaptation to mammals in the PB2 protein.

### 3.3 Humans

- Despite the widespread occurrence of HPAI A(H5N1) in wild birds, poultry, and some mammals in recent years, with many potential exposures of humans to infected animals, there have been no confirmed human cases of influenza A(H5N1) in the EU/EEA.
- The majority of human infections with avian influenza viruses reported since 1997 have been associated with unprotected exposure to poultry, live poultry markets, or contaminated environments. In addition, since March 2024, there have been 41 human cases of influenza A(H5) reported following exposure to dairy cattle infected with A(H5N1) virus. To date, there has been no evidence of sustained human-to-human transmission.
- During the reporting period from 28 February to 4 June 2026, six human cases of avian influenza A(H5N1) infection were publicly reported worldwide. All cases had direct or probable indirect exposure to poultry.
- During the same time period, one imported human case of avian influenza A(H9N2) was reported in Italy, representing the first human case of avian influenza A(H9N2) reported in the EU/EEA.
- With the extensive circulation of avian influenza viruses in bird populations globally, sporadic transmission to humans is likely to continue in settings where people have unprotected exposure to infected animals or their environment.

## 4 Options for response

The following sections on birds and mammals (4.1 and 4.2) include options for response for the coming months. Given the epidemiological trends observed during the current and previous reporting periods, the risk of HPAI A(H5) is expected to decline further this summer. General options for response included in previous reports are available in Annex D.

The sections on humans include all current options for response.

### 4.1 Birds

- Although the level of HPAI virus contamination of the environment is expected to be lower in the coming months, biosecurity measures in domestic bird establishments should be optimised and maintained at high level both during the production cycle and culling operations. This is particularly the case for establishments under the same ownership or located in densely populated poultry areas.
- While vaccination of poultry can substantially reduce the risk of outbreaks taking place in vaccinated compared to unvaccinated flocks, it does not provide complete protection on its own. Therefore, as it would be the case for unvaccinated flocks, vaccination should be accompanied by strict biosecurity measures and adequate surveillance.
- Surveillance in domestic birds should be maintained at a level that ensures early detection of infected poultry establishments. This is particularly the case for areas or production systems where secondary spread has been observed.
- It remains crucial to continuously monitor LPAI viruses of the A(H5) and A(H7) subtypes in wild and domestic birds, and introductions of these subtypes into poultry establishments, as these subtypes can mutate into their highly pathogenic forms once circulating in poultry.
- Given the detection of LPAI A(H9N2) virus of clade G5.5 in Hungary, as well as the endemicity of this particular clade in other countries outside Europe and its possible zoonotic potential, it is recommended to closely monitor its distribution and spread in Hungary and the rest of Europe, to report any further events involving the same virus, to characterise the virus, and to assess its risk of establishment in domestic birds.
- EFSA's Bird Flu Radar may be consulted to monitor the probability of HPAI virus introduction in wild bird populations over space and time, especially towards the end of summer: https://app.bto.org/hpai
- For wild birds, options for response include accurate and comprehensive recording of HPAI-associated mortality events (to estimate the impact on wild bird populations) and preventing disturbance of areas undergoing HPAI outbreaks to reduce virus spread (e.g. hunting activities, tourism, leisure activities, use of drones).

- Artificial feeding of wild birds should be avoided during high-risk periods to reduce the level of crowding of these species.
- Strengthening the genetic characterisation of viruses is recommended to quickly identify molecular markers of adaptation to mammals, which could indicate potential virus circulation in mammals and subsequent mammal-to-avian transmission. Viruses carrying such mutations may have an increased zoonotic potential and their biological characteristics should be further evaluated to assess the actual impact of the acquired mutations. To serve as an effective early warning system, sequences should be generated and shared in public databases as promptly as possible. Genetic data are also instrumental to track the virus spread, support epidemiological investigations in the distinction between primary and secondary outbreaks, and identify novel incursions of viruses that may represent a threat for human or animal health.

### 4.2 Mammals

- In the coming months, the risk of involvement of mammals in HPAI virus circulation is expected to be low. Nevertheless, surveillance of HPAI viruses in wild (e.g. red foxes) and free-roaming domestic carnivores (e.g. cats and dogs), as well as in farmed mammals in high-risk areas or establishments (e.g. zoos and mixed-species establishments), remains important.
- Research to investigate the role of mammals in maintaining HPAI viruses and driving their evolutionary dynamics is recommended.
- Pets and other captive mammals should not be fed with raw meat, raw pet food, or other raw animal products (e.g. raw milk) from sources that have not been adequately controlled for possible HPAI virus contamination. The risk associated with feeding contaminated raw pet food (based on poultry) to domestic cats, a practice that has been reported in several countries in the world, including in the EU, stresses the importance of highly sensitive surveillance systems for early detection, and of the removal and destruction of infected poultry flocks as well as of contaminated animal products.
- In high-risk areas, private veterinarians should be adequately informed and encouraged to participate in the surveillance of potential spillover events into pet carnivores such as domestic cats or dogs. If suspect clinical signs are observed (e.g. neurological or respiratory signs), these animals should be tested for HPAI.
- More accurate and timely reporting of HPAI virus detections in mammals is recommended in a way that reliable numbers of infected animals could be used as quantitative information for risk assessment.

### 4.3 Humans

- The risk of human exposure to avian influenza viruses can be reduced by implementing some of the preventive measures suggested below:
    - Use of appropriate personal protective equipment when in contact with potentially infected animals or highly contaminated environments.
    - Implementation of adequate biosecurity and biosafety measures at occupational sites where there is an increased risk of exposure in order to reduce the risk of zoonotic infection and ensure safe handling of potentially contaminated biological materials.

- Provision of information and guidance to raise awareness among people at risk of exposure and indicate how the risk can be mitigated. Guidance should be tailored to specific occupational groups, or individuals engaged in recreational activities where additional measures may be beneficial. Recommendations for personal protective measures should consider the working environment, tasks performed, routes of exposure, and environmental factors.
- Recommendations to the general public and those keeping backyard or commercial poultry are to avoid contact with sick or dead birds and wild animals. If dead animals are found, the relevant authorities should be informed in order to ensure their safe removal and further investigation, as needed.
- Detections of HPAI in apparently healthy wild ducks (EFSA and EURL, 2025) highlight the importance of precautionary measures when handling wild birds, even in the absence of clinical signs. Information to increase awareness of the risk of infection from animals and appropriate preventive measures should also be made available to those in contact with wild birds, such as hunters and wildlife workers.

- People exposed to animals with suspected or confirmed avian influenza virus infection while not wearing appropriate personal protective equipment should be monitored for symptoms for 10–14 days after the last exposure and tested if symptoms develop. Asymptomatic individuals exposed to animals infected with avian influenza virus should be assessed on a case-by-case basis and tested depending on the level of exposure. Further information on testing, follow-up and management of individuals with exposure and confirmed infection can be found in ECDC's guidance 'Testing and detection of zoonotic influenza virus infections in humans' (ECDC, 2022); 'Investigation protocol for human exposures and cases of avian influenza' (ECDC, 2023), and the ECDC/EFSA guidance 'Coordinated One Health investigation and management of outbreaks in humans and animals caused by zoonotic avian influenza viruses' (ECDC and EFSA, 2025).
- Countries should remain vigilant for potential human cases of avian influenza, especially in geographical areas where the virus is known to circulate in poultry, wild birds or other animals.
- In areas with ongoing avian influenza outbreaks in animals, healthcare workers (including primary care providers) should be made aware of the epidemiological situation in animal populations and the range of symptoms associated with avian influenza infection in humans (e.g. symptoms of upper or lower respiratory tract infection, but also non-respiratory symptoms, such as conjunctivitis, gastrointestinal or neurological symptoms).
- In the summer months between seasonal influenza periods, people seeking medical care who have symptoms compatible with zoonotic avian influenza infection should be asked about exposure to animals within two weeks of symptom onset, particularly in geographical areas with ongoing outbreaks in animals. Additional details on the strengthening of surveillance in the influenza inter-seasonal period are provided in ECDC's report 'Enhanced influenza surveillance to detect avian influenza virus infections in the EU/EEA during the inter-seasonal period' (ECDC, 2024).
- Detections of human cases of zoonotic avian influenza through surveillance systems for seasonal influenza highlight the importance of typing and subtyping samples. All influenza-positive specimens collected through seasonal influenza sentinel surveillance from both primary and secondary care sources should be typed and subtyped.
- Genetic changes in avian influenza viruses that may alter their zoonotic potential (increase capacity to infect humans or increase transmissibility) or their susceptibility to antivirals available to treat humans should be monitored (EFSA AHAW Panel and ECDC,

2025). Avian influenza viruses detected in humans should be sequenced and the sequence shared in public databases in a timely manner.

- Vaccination against seasonal influenza should be offered to individuals who are occupationally exposed to avian influenza viruses to limit infection with seasonal influenza in humans and reduce the risk of co-infection with human and avian influenza subtypes and the possibility of genetic reassortment between viruses (EFSA AHAW Panel and ECDC, 2025).
- Zoonotic avian influenza A(H5) vaccination in individuals occupationally or otherwise routinely exposed to infected animals or contaminated environments could be considered as a complementary preventive measure. While data on immune response induction are available, there is limited information on reduction in infection or onward transmission and protection against severe clinical disease (EFSA AHAW Panel and ECDC, 2025). Consequently, at present there is insufficient evidence to support a broad recommendation for their use in individuals occupationally or otherwise routinely exposed to infected animals across all EU/EEA countries (ECDC, 2025).
- The options for response are based on current available evidence, the epidemiological situation and the risk assessment for the EU/EEA. Recommended measures may need to be adapted if the epidemiological situation changes, or new evidence becomes available.

## Appendix A – Terms of Reference of the joint EFSA-ECDC mandate (M-2024-00009) accepted in March 2024

### A.1. Background and Terms of Reference as provided by the requestor

Avian influenza is an infectious viral disease in birds, including domestic poultry. Infections with avian influenza viruses in poultry cause two main forms of that disease that are distinguished by their virulence. The low pathogenic (LPAI) form generally only causes mild symptoms, while the highly pathogenic (HPAI) form results in very high mortality rates in most poultry species. That disease may have a severe impact on the profitability of poultry farming.

Avian influenza is mainly found in birds, but under certain circumstances infections can also occur in humans even though the risk is generally very low.

More than a decade ago, it was discovered that virus acquired the capability to be carried by wild birds over long distances. This occurred for the HPAI of the subtype A(H5N1) from South-East and Far East Asia to other parts of Asia, Europe and Africa as well as to North America. In the current epidemic the extent of the wild bird involvement in the epidemiology of the disease is exceptional.

The evolution of the HPAI epidemiological situation with high number of birds and new mammalian species affected is prompting response by both animal health and public health authorities in EU Member States and indicates the need for enhanced preparedness and prevention. Given the mammalian adaptation mutations detected in certain circulating viruses, the infection of mammals in fur farms, as well as an outbreak amongst cats extending the animal/human interface along with the suspicion of events of mammal-to-mammal transmission, animal health and public health authorities are currently working on addressing these challenges. In that context, they are developing or adapting their tools for epidemiological investigations, strengthening their collaboration issuing new emergency national legislations as well as adapting their surveillance guidance and programmes in the light of the upcoming seasonal flu season. The situation and actions above have been taken in reaction to:

- ECDC's current risk assessment that focuses on the immediate risk of avian influenza for human health fulfilling ECDC's new mandate and the Regulation (EU) 2022/2371[12] aiming to prevent and prepare for cross-border health threats, including epidemics; and
- EFSA's latest scientific opinions and reports coupled by measures taken (i.e., surveillance, prevention and control measures) under the Animal Health Law (i.e., Commission Delegated Regulation (EU) 2020/689[13] and Commission Delegated Regulation (EU) 2020/687[14]).

[12] Regulation (EU) 2022/2371 of the European Parliament and of the Council of 23 November 2022 on serious cross-border threats to health and repealing Decision No 1082/2013/EU. OJ L 314, 6.12.2022, p. 26–63.
[13] Commission Delegated Regulation (EU) 2020/689 of 17 December 2019 supplementing Regulation (EU) 2016/429 of the European Parliament and of the Council as regards rules for surveillance, eradication programmes, and disease-free status for certain listed and emerging diseases. OJ L 174, 3.6.2020, p. 211–340.
[14] Commission Delegated Regulation (EU) 2020/689 of 17 December 2019 supplementing Regulation (EU) 2016/429 of the European Parliament and of the Council as regards rules for surveillance, eradication programmes, and disease-free status for certain listed and emerging diseases. OJ L 174, 3.6.2020, p. 211–340.

In view of the One Health nature of this subject, and pursuant to Article 3 of Regulation (EU) 2022/2370[15] and Articles 29 and 31 of Regulation (EC) No 178/2002[16], the European Centre for Disease Prevention and Control (ECDC) and the European Food Safety Authority (EFSA) are requested to carry out the following task:

To provide regular quarterly scientific reports, updating on the avian influenza situation within the Union and worldwide, to the Commission by:

1 Analysing the epidemiological data on HPAI and LPAI from Member States and describe the evolution of virus spread from certain regions towards the EU and in case of significant changes in the epidemiology of avian influenza;
2 Analysing the temporal and spatial pattern of HPAI and LPAI, as appropriate, in poultry, captive and wild birds, kept and wild mammals, as well the risk factors involved in the occurrence, spread and persistence in the EU of the avian influenza virus in and at the interface of these animal populations with specific attention to zoonotic risks;
3 Describing the options for adapting preparedness, prevention, and control measures, based on the finding from point 1) and 2).

### A.2. Interpretation of the Terms of Reference

In reply to the Terms of Reference above, this Scientific Report gives an overview of the HPAI virus detections in poultry, captive and wild birds, as well as in mammals, in Europe and worldwide between 28 February and 4 June 2026, as reported by Member States and third countries via ADIS or WOAH-WAHIS. In addition, LPAI virus detections of specific relevance are included, and possible actions for preparedness in the EU are discussed based on the situation worldwide. Member States and other European countries where HPAI outbreaks have occurred in poultry submitted additional epidemiological data to EFSA, which have been used to analyse the characteristics of the affected poultry establishments.

However, it was not possible to collect data for a comprehensive risk factor analysis on the occurrence and persistence of HPAI viruses within the EU. Risk factor analysis requires not only case-related information but also data on the susceptible population (e.g. location of establishments and population structure), which should be collected in a harmonised manner across the EU. Limitations in data collection, reporting and analysis were explained in the first avian influenza overview report (EFSA, ECDC and EURL, 2017).

This report mainly describes information that has become available since the publication of the EFSA report for the period 29 November 2025 to 27 February 2026 (EFSA, ECDC and EURL, 2025c) and that might affect the interpretation of risks related to avian influenza introduction and/or spread in Europe.

[15] Regulation (EU) 2022/2370 of the European Parliament and of the Council of 23 November 2022 amending Regulation (EC) No 851/2004 establishing a European centre for disease prevention and control. OJ L 314, 6.12.2022, p. 1–25.
[16] Regulation (EC) No 178/2002 of the European Parliament and of the Council of 28 January 2002 laying down the general principles and requirements of food law, establishing the European Food Safety Authority and laying down procedures in matters of food safety. OJ L 31, 1.2.2002, p. 1–24.

## Appendix B – Data and Methodologies

### B.1. Data on animals

#### B.1.1. Overview of avian influenza outbreaks in Europe

For this report, data on HPAI outbreaks reported in Europe between 28 February and 4 June 2026 and submitted by Member States and other European countries via ADIS, were taken into account. Data extraction was carried on 4 June 2026. WOAH-WAHIS was consulted to complement the information for European countries not notifying HPAI via ADIS (United Kingdom (excluding Northern Ireland)). Additionally, countries affected by HPAI were asked to provide EFSA with more detailed epidemiological information on HPAI outbreaks in poultry. This information included details on the poultry species and production systems affected by HPAI, which were supplied in form of additional variables to complement the data reported via ADIS. All data collected are provided in Annex B. Since summer 2023, reporting countries in Europe were moreover requested to report any HPAI virus detections in mammals directly to EFSA. All data collected in the current reporting period are summarised in Annex C. Finally, the information European countries affected by avian influenza presented to the Standing Committee on Plants, Animals, Food and Feed (SCOPAFF), and the evidence on avian influenza outbreaks provided in the info notes from affected countries to the EC, were consulted to extract relevant information reported in Section 2.1.1. The presentations delivered at the SCOPAFF meetings are available on the EC website (European Commission, online).

Wild bird species have been categorised according to Table A.3, and the common and scientific names of wild bird species described in this report for Europe are reported in Table A.4 (both in Annex A). For mammals, the common and scientific names of the species involved are listed in Table 3. GISAID's EpiFlu™ database was accessed to download newly released avian influenza virus sequences.

The annexes to this Scientific Report are available here: https://doi.org/10.5281/zenodo.20920683

#### B.1.2. Overview of avian influenza outbreaks in other countries not reporting via ADIS

Data from WOAH-WAHIS on HPAI A(H5) and A(H7) in domestic and wild birds were used to describe and map the geographic distribution of HPAI virus detections in domestic and wild birds in other regions of the world based on their observation dates. Data were retrieved from WOAH-WAHIS and extracted on 4 June 2026. They were used and reproduced with permission. WOAH bears no responsibility for the integrity or accuracy of the data contained therein, not limited to: any deletion, manipulation, or reformatting of data that may have occurred beyond its control.

#### B.1.3. Genetic characterisation of avian influenza viruses: description of the nomenclature of the HPAI A(H5) viruses used in the document

The HA gene of clade 2.3.4.4 A(H5) viruses has rapidly evolved since the most recent official update of the nomenclature of the A/goose/Guangdong/1/1996-lineage H5Nx virus (Smith et al., 2015). This clade emerged in China in 2008 and since then has acquired various neuraminidase subtypes, including N1, N2, N3, N4, N5, N6, and N8, by reassortments with other avian influenza viruses from different regions, and has evolved into several subgroups. While a revised nomenclature of clade 2.3.4.4 viruses is pending, in previous reports the genetic clustering described in 2018 by Lee and co-authors, who recognised four groups (a–d) within clade 2.3.4.4, was used (Lee et al., 2018). Recently, an update to the unified nomenclature for clade 2.3.4.4 A(H5) viruses has been proposed by the WHO, and eight genetic groups (a–h) have been recognised. To align the nomenclature system between international organisations, this classification has been adopted for this report. Based on this proposed clustering, A(H5) viruses of clades 2.3.4.4a and d–h have mainly been circulating in poultry in Asia, while clades 2.3.4.4b and 2.3.4.4c have spread globally through wild bird migrations during 2014–2015 (2.3.4.4c) and from 2016 to the present day (2.3.4.4b). A list with the distribution of the different genetic clades reported by countries globally from birds, humans and the environment has been published by WHO in February 2023 (WHO, 2023).

### B.2. Data on humans

Data on the number of human cases caused by infection with avian influenza viruses were collected by ECDC. As part of epidemic intelligence activities at ECDC, multiple sources are scanned regularly to collect information on laboratory-confirmed human cases. Data were extracted and line lists developed to collect case-based information on virus type, date of disease onset, country of reporting, country of exposure, sex, age, exposure, clinical information (hospitalisation, severity), and outcome. All cases included in the line list and mentioned in the document have been laboratory-confirmed. Data are continuously checked for double entries and validity. The data on human cases cover the full period since the first human case was reported. Therefore, data on human cases refer to different time periods. Relevant information on human infections, risk factors, and the results from studies on infection and transmission with relevance for human health are included.

#### B.2.1. Method for phylogenetic and mutation analysis

The GISAID's EpiFlu™ Database (Shu and McCauley, 2017) was accessed on 22 June 2026 to retrieve sequences from human cases of avian influenza reported since 28 February 2026. In cases where the same strain had multiple submissions, the ones with the most complete segment data were chosen. Mutations were retrieved using FluMut (Github izsvenezie-virology, online-a, b) with the FluMutDB v6.4 mutation database (https://github.com/izsvenezie-virology/FluMutDB). For markers where FluMut reported an undefined amino acid, the marker received the annotation insufficient segment. Mutations found in less than 10% of the background sequences were considered significant. Clade information was retrieved from GISAID or assigned according to specified literature. Genin2 (https://github.com/izsvenezie-virology/genin2) and GenoFLU (Youk et al., 2023) were used to assign genotypes.

## Annex A – Data on HPAI virus detections in birds

Annex A includes additional figures and tables on HPAI virus detections in birds. The Annex is available on the EFSA Knowledge Junction community on Zenodo at: https://doi.org/10.5281/zenodo.20920683

## Annex B – Characteristics of the HPAI-affected poultry establishments

Annex B includes the HPAI outbreaks in poultry for which additional data on the characteristics of the affected poultry establishments were collected and provided by reporting countries in Europe during the current reporting period. It also includes data on outbreaks that occurred too close to the publication of the previous report and are therefore included this time. The Annex is available on the EFSA Knowledge Junction community on Zenodo at: https://doi.org/10.5281/zenodo.20920683

## Annex C – Data on HPAI virus detections in mammals

Annex C includes data on HPAI virus detections in mammals for which additional data were collected and provided by reporting countries in Europe during the current reporting period. The Annex is available on the EFSA Knowledge Junction community on Zenodo at: https://doi.org/10.5281/zenodo.20920683

## Annex D – General options for response in animals

Annex D contains general options for response in animals that are valid throughout the year, irrespective of the current reporting period.

The Annex is available on the EFSA Knowledge Junction community on Zenodo at: https://doi.org/10.5281/zenodo.20920683

## Annex E – Acknowledgements

All genome sequences and associated metadata linked in the below dataset are published in GISAID's EpiFlu™ database (Shu and McCauley, 2017). To view the contributors of each individual sequence with details such as accession number, virus name, collection date, originating and submitting lab, and the list of authors, visit: https://doi.org/10.55876/gis8.260624dr and https://doi.org/10.55876/gis8.260622cr

The EFSA Journal is a publication of the European Food Safety Authority, an agency of the European Union.
